# Semaphorin 3C promotes *de novo* steroidogenesis in prostate cancer cells

**DOI:** 10.1530/ERC-23-0010

**Published:** 2023-11-06

**Authors:** Parvin Yenki, Satyam Bhasin, Liang Liu, Noushin Nabavi, Chi Wing Cheng, Kevin J Tam, James W Peacock, Hans H Adomat, Tabitha Tombe, Ladan Fazli, Larissa Ivanova, Christopher Dusek, Shahram Khosravi, Emma S Tomlinson Guns, Yuzhuo Wang, Ralph Buttyan, Martin E Gleave, Christopher J Ong

**Affiliations:** 1 The Vancouver Prostate Centre, Vancouver General Hospital, Vancouver, BC, Canada; 2 Department of Urologic Sciences, University of British Columbia, Vancouver, BC, Canada

**Keywords:** castration-resistant prostate cancer (CRPC), semaphorin 3C (SEMA3C), receptor tyrosine kinase (RTK), steroidogenesis, intratumoral androgen synthesis, androgen receptor (AR)

## Abstract

Intratumoral androgen biosynthesis contributes to castration-resistant prostate cancer progression in patients treated with androgen deprivation therapy. The molecular mechanisms by which castration-resistant prostate cancer acquires the capacity for androgen biosynthesis to bypass androgen deprivation therapy are not entirely known. Here, we show that semaphorin 3C, a secreted signaling protein that is highly expressed in castration-resistant prostate cancer, can promote steroidogenesis by altering the expression profile of key steroidogenic enzymes. Semaphorin 3C not only upregulates enzymes required for androgen synthesis from dehydroepiandrosterone or *de novo* from cholesterol but also simultaneously downregulates enzymes involved in the androgen inactivation pathway. These changes in gene expression correlate with increased production of androgens induced by semaphorin 3C in prostate cancer model cells. Moreover, semaphorin 3C upregulates androgen synthesis in LNCaP cell-derived xenograft tumors, likely contributing to the enhanced *in vivo* tumor growth rate post castration. Furthermore, semaphorin 3C activates sterol regulatory element-binding protein, a transcription factor that upregulates enzymes involved in the synthesis of cholesterol, a sole precursor for *de novo* steroidogenesis. The ability of semaphorin 3C to promote intratumoral androgen synthesis may be a key mechanism contributing to the reactivation of the androgen receptor pathway in castration-resistant prostate cancer, conferring continued growth under androgen deprivation therapy. These findings identify semaphorin 3C as a potential therapeutic target for suppressing intratumoral steroidogenesis.

## Introduction

Despite its initial efficacy, androgen deprivation therapy (ADT) remains a palliative treatment for metastatic prostate cancer, and patients eventually progress to castration-resistant prostate cancer (CRPC) (So *et al.* 2005, Tilki *et al.* 2016). CRPC cells are capable of intratumoral androgen synthesis despite the low serum testosterone during ADT (Mostaghel *et al.* 2007, Dillard *et al.* 2008, Montgomery *et al.* 2008), providing a sufficient level of androgens for the reactivation of the androgen receptor (AR) (Goldenberg *et al.* 1988, Bruchovsky *et al.* 1989).

The molecular pathways that underlie the activation of intratumoral androgen synthesis in PCa cells upon systemic androgen deprivation remain poorly understood. This study is a step toward unraveling the signaling programs that affect steroidogenesis in PCa cells.

Several lines of evidence suggest that activation of various receptor tyrosine kinase (RTK) pathways can stimulate steroidogenesis. For instance, EGF and insulin-like growth factor (IGF) 1 stimulate steroidogenesis in Leydig cells (Sordoillet *et al.* 1991, Evaul & Hammes 2008). Furthermore, insulin and IGF2 have been shown to increase steroidogenesis in PCa cells (Lubik *et al.* 2011, 2013). Because of its therapeutic significance, the identification of other drivers of intratumoral steroidogenesis remains an important objective in the study of CRPC.

Semaphorin 3C (SEMA3C) is a signaling protein that acts as an autocrine growth factor that activates various RTKs, such as EGFR, HER2, and MET, in a cognate ligand-independent manner. Also, evidence shows that SEMA3C can promote PCa growth in androgen-deprived conditions (Peacock *et al.* 2018). In addition, SEMA3C has been shown to play a role in perineural invasion of prostate cancer cells (Maru *et al.* 2001, DeLancey *et al.* 2013, Ciftci *et al.* 2015, Kraus *et al.* 2019, Yin *et al.* 2021). However, the functional role of SEMA3C in promoting castration-resistant growth of PCa cells remains unclear.

Herein, we discovered that SEMA3C stimulates androgen biogenesis from dehydroepiandrosterone (DHEA) or *de novo* from cholesterol in multiple PCa cell lines and our xenograft LNCaP model. This phenomenon is accompanied by the upregulation of multiple steroidogenic enzymes while suppressing the expression of androgen-catabolizing enzymes by SEMA3C. Furthermore, activation of sterol regulatory element-binding protein (SREBP) transcription factors and induction of cholestrogenic enzymes by SEMA3C are consistent with increased intratumoral cholesterol levels by SEMA3C in xenograft LNCaP tumors post castration. Notably, erlotinib, the U.S. Food and Drug Administration-approved epidermal growth factor receptor (EGFR) inhibitor, attenuates the induction of steroidogenic enzymes by SEMA3C, suggesting EGFR mediation in stimulating androgen biosynthesis by SEMA3C. We propose to investigate SEMA3C as a potential therapeutic target that may play a role in promoting CRPC growth through intratumoral androgen biosynthesis, *de novo* from cholesterol, by triggering RTK signaling pathways.

## Materials and methods

### Cell culture and treatments

LNCaP and 22Rv1 cell lines, purchased from American Type Culture Collection, were authenticated by IDEXX BioAnalytics case #20201/21100-2014 (Columbia, MO, USA). C4-2 cells, kindly provided by Dr Leland W K Chung (MD Anderson Cancer Center, TX, USA), were authenticated by whole-genome and whole-transcriptome sequencing on Illumina Genome Analyzer IIx platform in 2009. LAPC4 cells, a kind gift from Dr Buttyan (Vancouver Prostate Centre, Vancouver, Canada) authenticated by IDEXX BioAnalytics case #27880-2018. All cell lines were maintained in RPMI 1640 media supplemented with 10% (v/v) fetal bovine serum with no antibiotics and were tested for mycoplasma contamination weekly, using a mycoplasma detection kit (InvivoGen, San Diego, CA, USA; Cat. No. rep-pt2).

To culture LAPC4 cells, plates were coated by incubation with poly-l-lysine 0.01% (Sigma, Cat. No. P4832) in phosphate-buffered saline (v/v, 1/20) for about 20 min before adding the medium. LNCaP and 22Rv1 cells were transduced with a lentiviral vector harboring full-length SEMA3C gene (LNCaP^SEMA3C^_,_ 22Rv1^SEMA3C^) or an empty vector (LNCaP^vector^_,_ 22Rv1^vector^) as described in Peacock *et al.* 2018. To generate recombinant SEMA3C, CHO cells were transduced by lentivirus expressing SEMA3C full-length gene, as previously described (Peacock *et al.* 2018). To knockdown SEMA3C gene, 22Rv1 and C4-2 cells were transfected with siSEMA3C #1 (Ambion, Cat. No. 4392420) or siSEMA3C #2 (Invitrogen, Cat. No. 10620319), and negative control siRNA (Ambion, s4390843) using RNAiMAX (Invitrogen, Cat. No. 13778-075). Thawed cells from −80℃ were passaged two times within a week prior to conduct experiments. Equal numbers of live cells were used. Live cells were quantified by trypan blue exclusion. Depleted cells from steroids with charcoal-stripped serum (CSS)-supplemented media 5% (v/v) (Thermo Fisher Scientific, Cat. No. 12676029) were treated with DHEA 1 mg/mL equivalent to 3.46 μM (Steraloids, Newport, RI, USA; Cat. No. A8500-000), or with ^14^C-acetate 6uCi/ml (PerkinElmer, Cat. No. NEC553050UC) for 48 h.

### RNA isolation and qPCR analysis

RNA was isolated from cells or tumor tissue using PureLink RNA Mini Kit (Life Technologies, Cat. No. 12183025), next was reverse transcribed by Superscript II (Invitrogen, Cat. No. 18064-014), using random hexamers (Roche, Cat. No. R15504) and dNTP (Thermo Fisher Scientific, Cat. No. R0192). qPCR reactions in three technical replicates were carried out on AB ViiA7 real-time PCR machine using Platinum SYBR Green (Invitrogen, Cat. No. 11744-500), then results were analyzed by ΔΔCt method using GAPDH as an endogenous control. qPCR primer sequences can be found in Supplementary Table 1 (see section on supplementary materials given at the end of this article).

### Next-generation comparative transcriptome sequencing

Total RNA from LNCaP^SEMA3C^ and LNCaP^vector^ (*n* = 3 per group) was isolated with Maxwell® RSC simply RNA Tissue Kit (Promega, Cat. No. AS1340) using Maxwell RSC Instrument (Promega, Cat. No. AS4500). Following quality control by TapeStation system, strand-specific RNA library was prepared using TruSeq mRNA library prep from Illumina using 300 ng of total RNA, quantified by Qubit RNA HS assay kit (Thermo Fisher Scientific, Cat. No. Q32852). After qPCR quantitation using KAPA Library quantification kit (KAPA Biosystems, Wilmington, MA, USA; Cat. No. KR0405), indexed RNA libraries were sequenced by NextSeq 500 high-throughput benchtop sequencer (Illumina) using 500/550 high output kit v2.5 kit (150 cycles, paired end (2 × 75 bp)) with target coverage of 20 million reads. Alignment onto the human reference genome (GRCh38.90) was performed using STAR (2.6.0c), and gene expressions are then estimated by HTSeq-count 0.10.0 (counting mode: intersection-strict). Differential expression of genes was assessed by DESeq2 1.16.1.

### Luciferase assay

Transient transfection of LNCaP cells with 1.2 µg ARR2PB-Luciferase reporter plasmid (a kind gift from Dr Rennie lab at Vancouver Prostate Centre, University of British Columbia) and SREBP-responsive element, SRE synthRE, (Active Motif, Carlsbad, CA, USA; Cat. No. 32192) was conducted using MirusIT-2020 transfection reagent (Mirus, Madison, WI, USA; Cat. No. MIR 5400). Cells were then treated with DHEA 3.4uM, and recombinant SEMA3C 1uM or vehicle for an additional 48 h prior to harvesting for luciferase assay using the Dual-Luciferase Reporter Assay System (Promega, Cat. No. E1960). Luminescence from all samples in technical duplicates was read on TECAN Infinite M200 PRO and was normalized to Renilla luciferase.

### Western blotting

Lysis buffer contained 50 mM Tris-HCl, 150 mM NaCl, 1% NP40, 10 mM NaF, 10% glycerol, and protease inhibitor cocktail (Roche, Cat. No. 04693116001). Western blots were imaged by a LI-COR Odyssey system. Loading controls were run on the same blots. Primary antibodies include AR (Santa Cruz Biotechnology, Cat. No. sc-816), PSA (Cell Signaling Technology, Cat. No. 5877), SEMA3C (Santa Cruz Biotechnology, Cat. No. sc-27796), AKR1C3 (Abcam, Cat. No. ab49680), HSD3B2 (Abnova, Taipei, Taiwan; Cat. No. 3284-mo2), HSD17B3 (Abnova, Cat. No. 29-119), FASN (Cell Signaling Technology, Cat. No. 3180), HMGCS1 (Cell Signaling Technology, Cat. No. 36877), SREBP-1 (Santa Cruz Biotechnology, Cat. No. sc-13551), UGT2B17 (Abcam, Cat. No. ab126269), and actin (Sigma-Aldrich, Cat. No. A2066). Secondary antibodies were anti-rabbit Alexa Fluor 680 (Invitrogen, Cat. No. A21109), anti-mouse Alexa Fluor 680 (Invitrogen, Cat. No. A21058), and anti-goat Alexa Fluor 680 (Invitrogen, Cat. No. A21084).

### Analysis of clinical datasets

UCSC Xena visualization software (https://xena.ucsc.edu/) was used to examine coexpression of SEMA3C and steroidogenic enzymes in the TCGA PRAD prostate cancer dataset. Fold-changes are shown in color, log2 (normalized_count + 1) mean is subtracted per column across 550 samples. cBioPortal plots of the TCGA provisional dataset (https://www.cbioportal.org/) show the degree of correlation in expression of SEMA3C and each of enzymes CYP11A1 and SRD5A2, and AR target FKBP5. Pearson’s and Spearman’s correlations are indicated.

### *In vivo* study

All animal procedures were conducted according to the standards and regulations of the Canadian Council on Animal Care and approved protocol (A15-0150) by the University of British Columbia Committee on Animal Care. To create LNCaP CRPC xenograft model (Peacock *et al.* 2018), 1 × 10^6^ LNCaP^SEMA3C^ and LNCaP^vector^ cells were subcutaneously inoculated with 0.1 mL Matrigel (Becton Dickinson Labware) in the flank region of 6‐ to 8‐week‐old male athymic nude mice (Jackson Laboratory) via a 27‐gauge needle under methoxyflurane anesthesia. Mice bearing LNCaP tumors were weekly monitored for body weight, tumor volume, and serum PSA levels. PSA concentration in sera samples obtained from tail vein incisions was determined by the Roche Diagnostics cobas e411 immunoassay system which is an automated, random access multichannel analyzer for immunological analysis using enhanced chemiluminescent technology. When tumor volumes or serum PSA levels reached over 200 mm^3^ or a minimum of 50 ng/mL, castration was performed via the scrotum under isoflurane anesthesia. The experimental endpoint was 4 weeks post castration when subcutaneous tumors were harvested to either snap-freeze in liquid nitrogen to maintain in −80℃ for RNA isolation and steroid analysis (*n* = 6 per group) or to fix in formalin to prepare tissue microarray. SEMA3C protein expression in LNCaP^SEMA3C^ and LNCaP^vector^ tumors were compared by immunohistochemistry (IHC) staining of formalin-fixed tissues with a discovery DAB Map detection kit.

### Lipid and steroid analysis

Steroids and lipid content were isolated from conditioned cell culture media, homogenates of collected cells or xenograft tumor tissues using solid phase extraction (SPE) columns. Isolated steroids or lipids were analyzed with liquid chromatography coupled with mass spectrometry (LC-MS). Details of these procedures are found in the Supplementary Materials.

### Statistical analysis

All results were presented as mean ± s.d. from at least three independent experiments. Data from biological replicates were analyzed using the two‐tail Student’s *t*-test and differences with *P* < 0.05 reached statistical significance.

## Results

### SEMA3C altered the expression of steroidogenic enzymes in PCa cells

**Figure 1:**
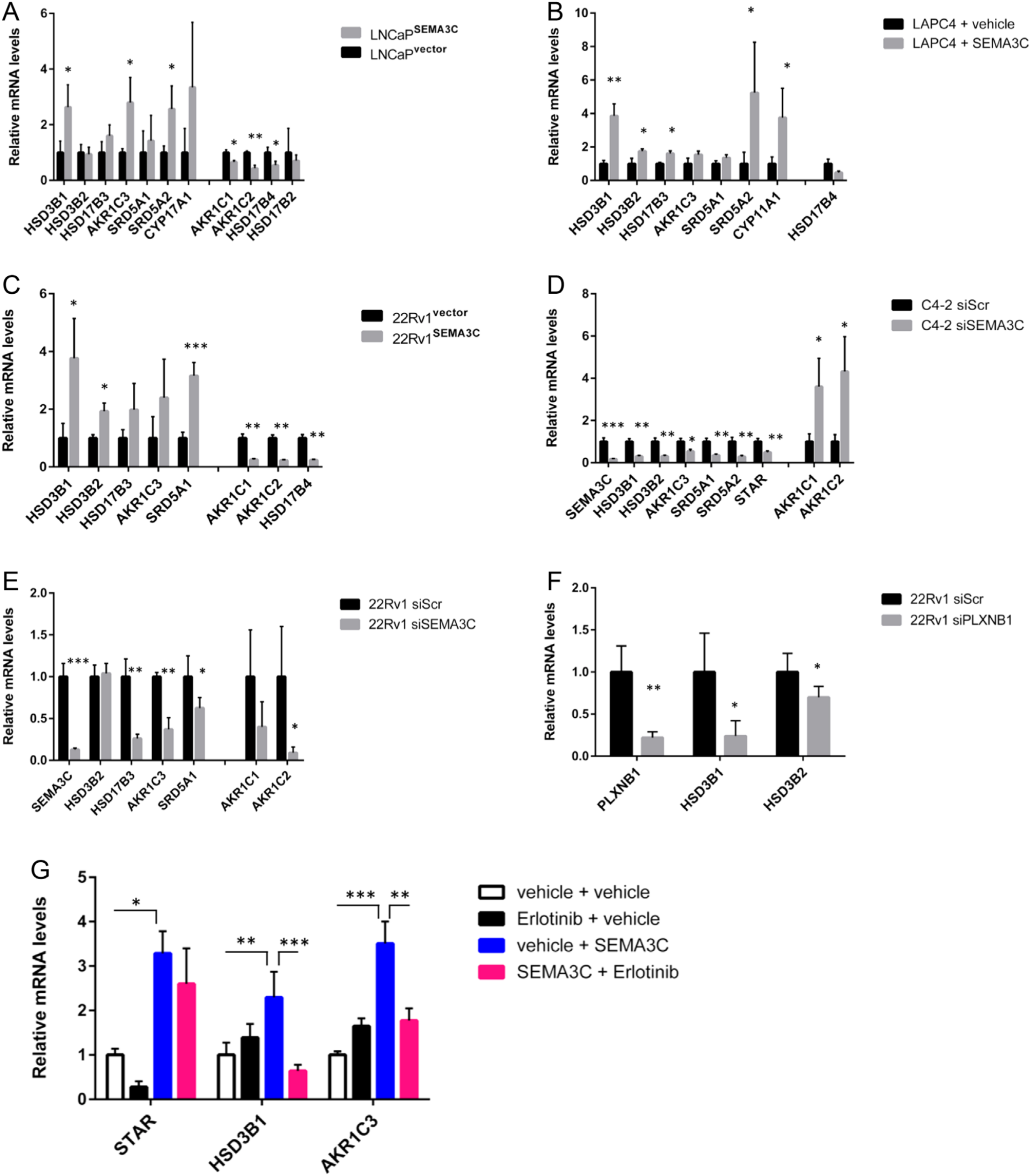
SEMA3C altered the expression of steroidogenesis pathway enzymes. qPCR was performed to quantify relative mRNA expression of a panel of steroidogenic enzymes in (A) LNCaP^SEMA3C^ vs LNCaP^vector^ cells, (B) recombinant SEMA3C (1 μM)-treated LAPC4 vs mock cells, and (C) 22Rv1^SEMA3C^ vs 22Rv1^vector^ cells. (D) mRNA expression of steroidogenic enzymes was compared between siSEMA3C-treated C4-2 and siScr-treated C4-2. (E) mRNA expression of steroidogenic enzymes was compared between siSEMA3C-treated 22Rv1 and siScr-treated 22Rv1. (F) mRNA expression of steroidogenic enzymes was quantified in siPLXNB1-treated 22Rv1 cells, compared to siScr-treated 22Rv1 cells. (G) LNCaP cells were treated with erlotinib 2 μM approximately 2 h prior to the treatment with recombinant SEMA3C 1 μM. After 48 h, mRNA expression of steroidogenic enzymes was analyzed by qPCR (mean ± s.d.; *P < 0.05, **P < 0.01, ***P < 0.001). A full color version of this figure is available at https://doi.org/10.1530/ERC-23-0010.

We evaluated the effects of SEMA3C to impact the expression profile of the steroidogenic enzymes in castration-sensitive LNCaP and LAPC4 cells and castration-resistant 22Rv1 and C4-2 cells. Comparison of the expression of a battery of steroidogenic genes between LNCaP^SEMA3C^ and LNCaP^vector^ cells showed that a statistically significant induction in mRNA expression of HSD3B1, AKR1C3, and SRD5A2 in the SEMA3C overexpressing cells. Increases of CYP17A1, HSD3B2, HSD17B3, and SRD5A1 did not reach statistical significance (Fig. 1A). SEMA3C overexpression was validated in LNCaP^SEMA3C^ (Appendix Fig. S1a). Also, we treated LNCaP cells with increasing concentrations of recombinant SEMA3C (0.5 and 1 μM) for 24 h. We found that mRNA expression of steroidogenic enzymes HSD3B1, HSD17B3 AKR1C3, and SRD5A2 increased in a dose-dependent manner (Appendix Fig. S1b). Similar to LNCaP cells, LAPC4 cells responded to recombinant SEMA3C treatment (1 μM) by significant induction of HSD3B1, HSD17B3, AKR1C3, and SRD5A2 along with CYP11A1 and HSD3B2 (Fig. 1B). SEMA3C expression is correlated with expression of steroidogenic enzymes CYP11A1 and SRD5A2 as well as the AR target gene, FKBP5, in prostate adenocarcinoma patients according to TCGA PanCancer dataset available on cBioPortal. Also, UCSC Xena confirmed coexpression of SEMA3C with FKBP5, CYP11A1, and SRD5A2 in prostate cancer patients (https://xena.ucsc.edu) (Appendix Fig. S1c).

SEMA3C overexpression in 22Rv1 cells resulted in the upregulation of multiple steroidogenic enzymes, including HSD3B1, HSD3B2, and SRD5A1. Induction of AKR1C3, HSD17B3 enzymes did not reach the statistical significance (Fig. 1C). SEMA3C overexpression was validated in 22Rv1 cells (Appendix Fig. S1d). On the contrary, SEMA3C inhibited the expression of androgen-catabolizing enzymes, AKR1C1, AKR1C2, and HSD17B4 in LNCaP (Fig. 1A) and 22Rv1 cells (Fig. 1C). Suppression of HSD17B2 in LNCaP (Fig. 1A) and HSD17B4 in LAPC4 cells (Fig. 1B) did not reach statistical significance.

Silencing SEMA3C resulted in reduced mRNA expression of HSD3B1, HSD3B2, AKR1C3, SRD5A1, and SRD5A2 along with STAR in C4-2 cells. Conversely, AKR1C1 and AKR1C2 mRNA expression was increased in siSEMA3C-treated C4-2 cells compared to those treated with siScrambled (siScr) (Fig. 1D). However, SEMA3C silencing in 22Rv1 cells only resulted in downregulation of HSD17B3, AKR1C3, and SRD5A1 enzymes and also showed suppression of AKR1C1 and AKR1C2 (Fig. 1E). SEMA3C knockdown was validated in 22Rv1 and C4-2 cells (Appendix Fig. S1e). PLXNB1 is a receptor for SEMA3C, so we investigated whether silencing of this SEMA3C receptor can attenuate the effect of SEMA3C on the expression of steroidogenic enzymes. siRNA-mediated silencing of PLXNB1 in 22Rv1 cells resulted in the reduced mRNA expression of both isoforms of HSD3B enzyme (Fig. 1F). To study the potential role of EGFR pathway in the stimulation of steroidogenesis pathway by SEMA3C, we inhibited EGFR activity with erlotinib prior to recombinant SEMA3C treatment of LNCaP cells. EGFR inhibition attenuated the induction of STAR, HSD3B1, and AKR1C3 by SEMA3C in LNCaP cells (Fig. 1G). EGFR inhibition by erlotinib was confirmed by measuring phosphorylated and total EGFR and MAPK by immunoblotting (Appendix Fig. S1f).

### SEMA3C increased androgen synthesis from adrenal precursor, DHEA

**Figure 2:**
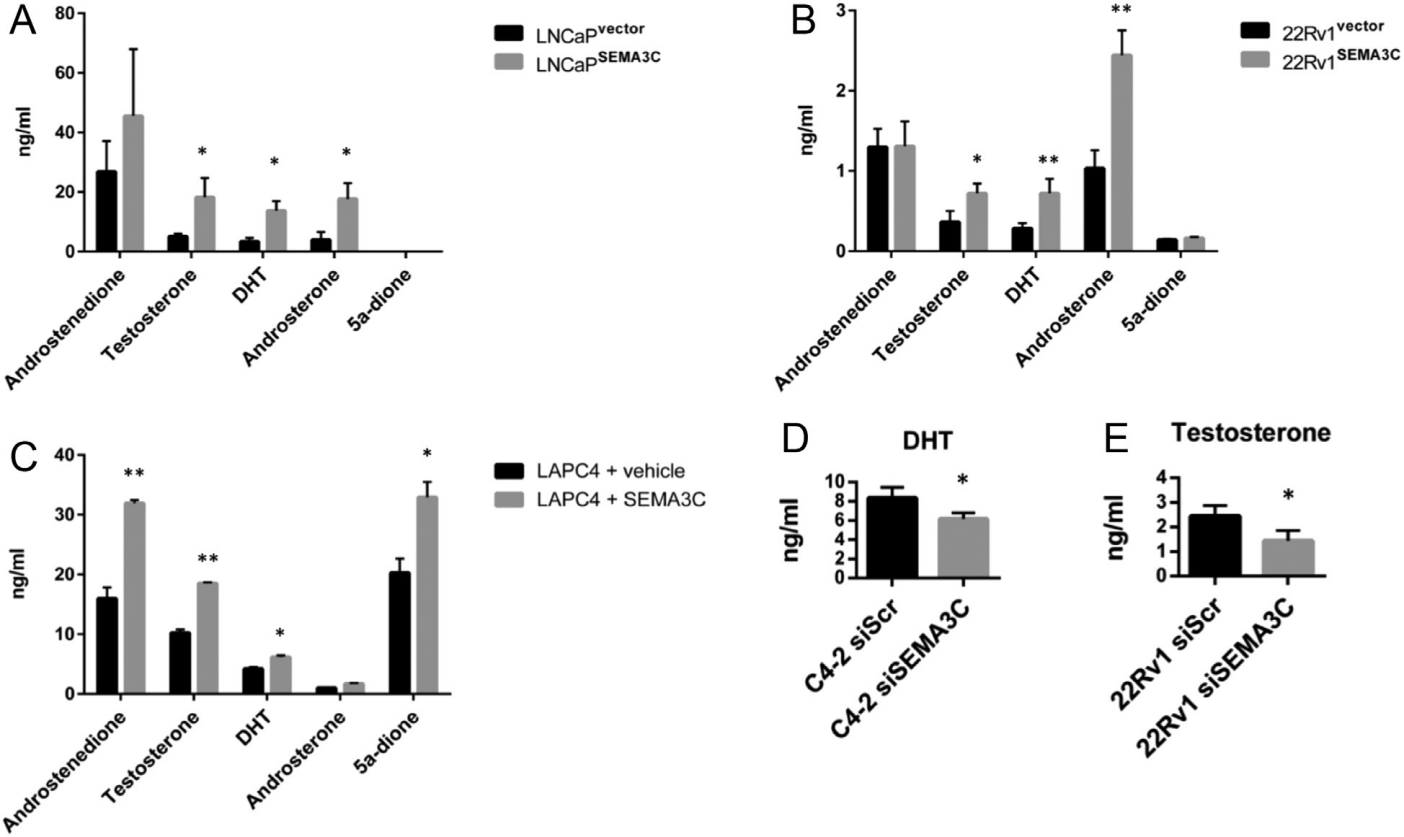
SEMA3C affected steroid synthesis in PCa cells. (A) LNCaP^SEMA3C^ and LNCaP^vector^ cells treated with DHEA 3.4 μM in 5% CSS-supplemented phenol red-free RPMI for 48 h. The concentration of a panel of steroids in the conditioned media derived from treated cells was analyzed by LC-MS. (B) Identical treatment for 22Rv1^SEMA3C^ vs control cells was followed by an LC-MS analysis to measure the concentration of steroids in the conditioned media. (C) Steroids which were isolated from the conditioned media derived from LAPC4 cells treated with recombinant SEMA3C (1 μM) or vehicle in the presence of DHEA (3.4 μM) for 48 h were analyzed by LC-MS. The concentration of steroids was analyzed in (D) C4-2 and (E) 22Rv1 cells following siRNA-mediated silencing of SEMA3C, in the presence of DHEA (3.4 μM). (*n* = 3, mean ± s.d.; *P < 0.05, **P < 0.01).

Since ADT does not reduce DHEA serum level, it is the most abundant steroid hormone in the serum of PCa patients post ADT. Cancer cells may use this adrenal precursor as a substrate for intratumoral androgen synthesis (Nishiyama *et al.* 2004, Labrie 2010, Narimoto *et al.* 2010, Arai *et al.* 2011). To assess the effect of SEMA3C on the androgen biosynthesis in various PCa cell lines, we incubated cells with DHEA in CSS-supplemented media for 48 h. SEMA3C overexpression resulted in increased testosterone, dihydrotestosterone (DHT), androsterone released by LNCaP cells (Fig. 2A). Also, the intracellular concentration of the same androgens within LNCaP^SEMA3C^ cells was significantly higher compared to LNCaP^vector^ cells (Appendix Fig. S2a). Similarly, SEMA3C overexpression in 22Rv1 cells increased secreted (Fig. 2B) and intracellular (Appendix Fig. S2b) levels of testosterone, DHT and androsterone. Also, LC-MS analysis of steroids isolated from the conditioned media derived from LAPC4 cells treated with recombinant SEMA3C showed comparable results with SEMA3C-overexpressing LNCaP and 22Rv1 cells. However, the increase of androstenedione reached statistical significance, and SEMA3C caused an increase in the 5a-dione level instead of androsterone (Fig. 2C). Silencing SEMA3C suppressed the production of DHT in C4-2 cells (Fig. 2D) and testosterone in 22Rv1 cells (Fig. 2E). In order to rule out a contribution from proliferation, we used PrestoBlue assay to evaluate the proliferative effects of SEMA3C overexpression in LNCaP and 22Rv1 cells and SEMA3C knockdown in C4-2 and 22Rv1 cells, as well as recombinant SEMA3C treatment on LAPC4 cells. We found that at early time points up to 48 h, SEMA3C did not significantly alter the proliferation of PCa cells (Appendix Fig. S2c). Consistent with these findings, Peacock *et al.* results showed that the difference between the proliferation rate of SEMA3C-overexpressing LNCaP and empty LNCaP cells did not reach statistical significance sooner than day 5 (Peacock *et al.* 2018). These data suggest that the impact of SEMA3C on steroid production during the first 48 h is likely not due to SEMA3C’s effect on the proliferation of PCa cells.

### SEMA3C increased AR-dependent gene expression

**Figure 3:**
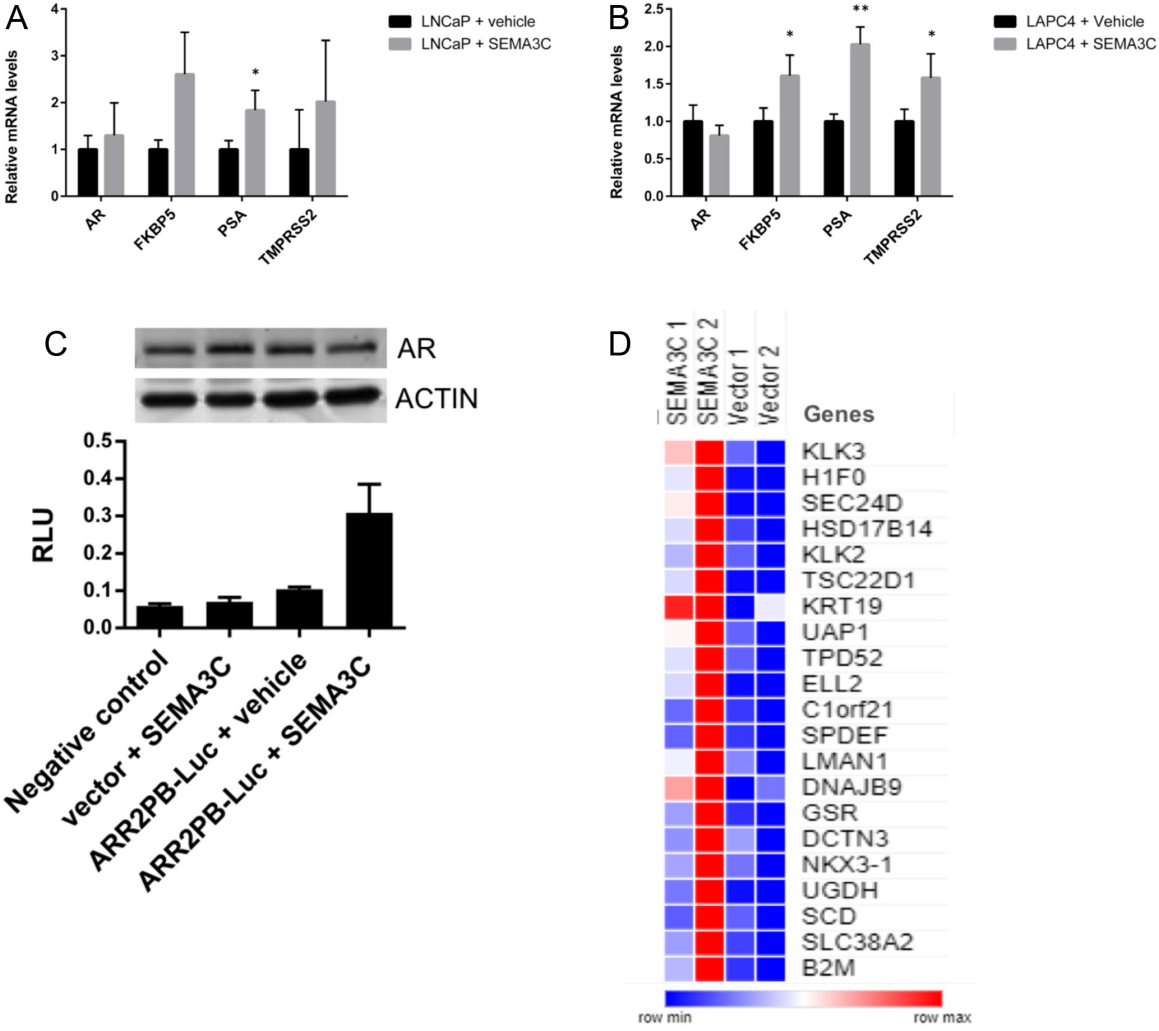
SEMA3C induces AR-dependent gene expression. Recombinant SEMA3C 1 μM treatment of (A) LNCaP and (B) LAPC4 cells, supplemented with DHEA 3.4 μM for 24 h upregulated mRNA expression of AR target genes, quantified by RT-qPCR method. (C) LNCaP cells were transiently transfected with ARR2PB reporter plasmid and then were treated with recombinant SEMA3C 1 μM or vehicle in DHEA (3.4 μM)-supplemented media. Luciferase assay showed SEMA3C signaling increased probasin promoter activity compared to vehicle. The whole protein of the treated cells was immunoblotted for AR protein level. (D) Total RNA was isolated from LNCaP^SEMA3C^ and LNCaP^vector^ (*n* = 3 per group) to be analyzed by RNA-seq. This result shows the differential expression of a set of AR-regulated genes between LNCaP^SEMA3C^ and LNCaP^vector^ cells. (*n* = 3, mean ± s.d.; *P < 0.05, **P < 0.01).

We found that treatment with recombinant SEMA3C for 48 h increases mRNA expression of AR target genes, FKBP5, TMPRSS2, and PSA in LNCaP (Fig. 3A) and LAPC4 cells (Fig. 3B). Luciferase assay showed that recombinant SEMA3C treatment of LNCaP cells, transfected with a luciferase reporter plasmid with probasin promoter (ARR2PB), increased AR transcriptional activity (Fig. 3C). RNA-seq analysis in LNCaP^SEMA3C^ vs empty LNCaP found an increase in a number of AR-regulated genes. The differential expression of AR targets is presented as the heatmap (Fig. 3D).

### SEMA3C induced *de novo* steroid synthesis from cholesterol precursor

**Figure 4:**
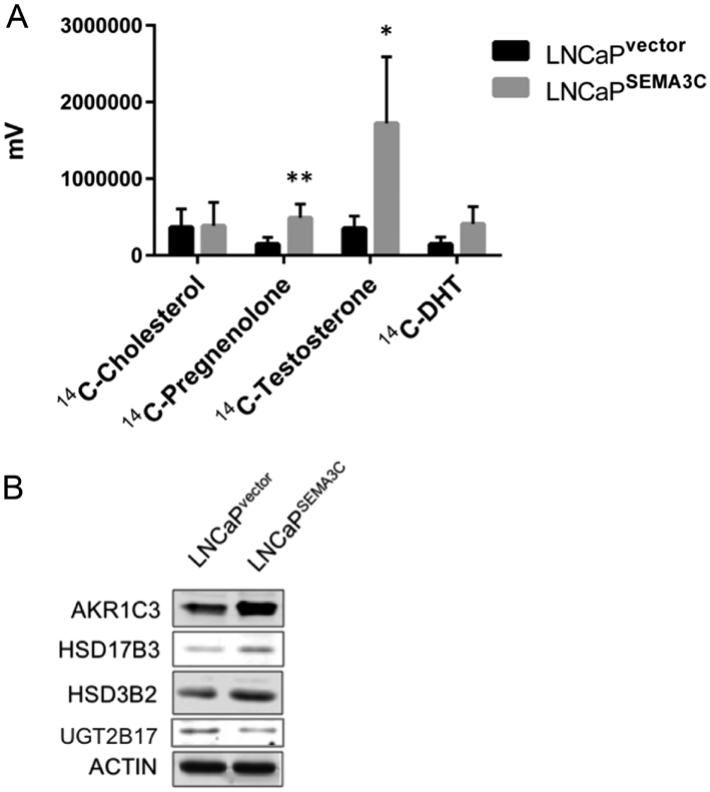
SEMA3C increases *de novo* steroidogenesis. (A) Steroids in media of LNCaP^SEMA3C^ and LNCaP^vector^ cells following 72 h treatment with 6 μCi/mL 14C-acetate in phenol red-free RPMI with 5% (v/v) CSS were identified and quantified by HPLC and radiometric detection with HPLC-coupled radiometric detection. (B) The whole protein of LNCaP^SEMA3C^ and LNCaP^vector^ was immunoblotted to examine the protein level of enzymes involved in steroidogenesis pathway. We found altered expression of AKR1C3, HSD3B17, HSD3B2, and UGT2B17. (*n* = 6, mean ± s.d.; *P < 0.05, **P < 0.01).

We assessed the ability of SEMA3C to increase *de novo* steroidogenesis by treating LNCaP^SEMA3C^ and LNCaP^vector^ cells with ^14^C-acetate in CSS-supplemented media for 48 h. Released steroids in the conditioned media were isolated with SPE columns and radiolabeled steroids were identified and quantified by HPLC coupled with a radiometric Scintillation Analyzer. We used radioisotope-labeled controls including cholesterol, pregnenolone, and DHT to identify the MS peaks of ^14^C-labaeled steroids isolated from LNCaP conditioned media. We found that SEMA3C overexpression is correlated with a statistically significant increase in ^14^C-pregnenolone, ^14^C-testosterone, and ^14^C-DHT levels in LNCaP cells (Fig. 4A) (Appendix Fig. S3). Protein expression of a few enzymes in steroidogenesis enzymes was altered by SEMA3C overexpression in LNCaP cells (Fig. 4B).

### SEMA3C induced SREBP-regulated genes

**Figure 5:**
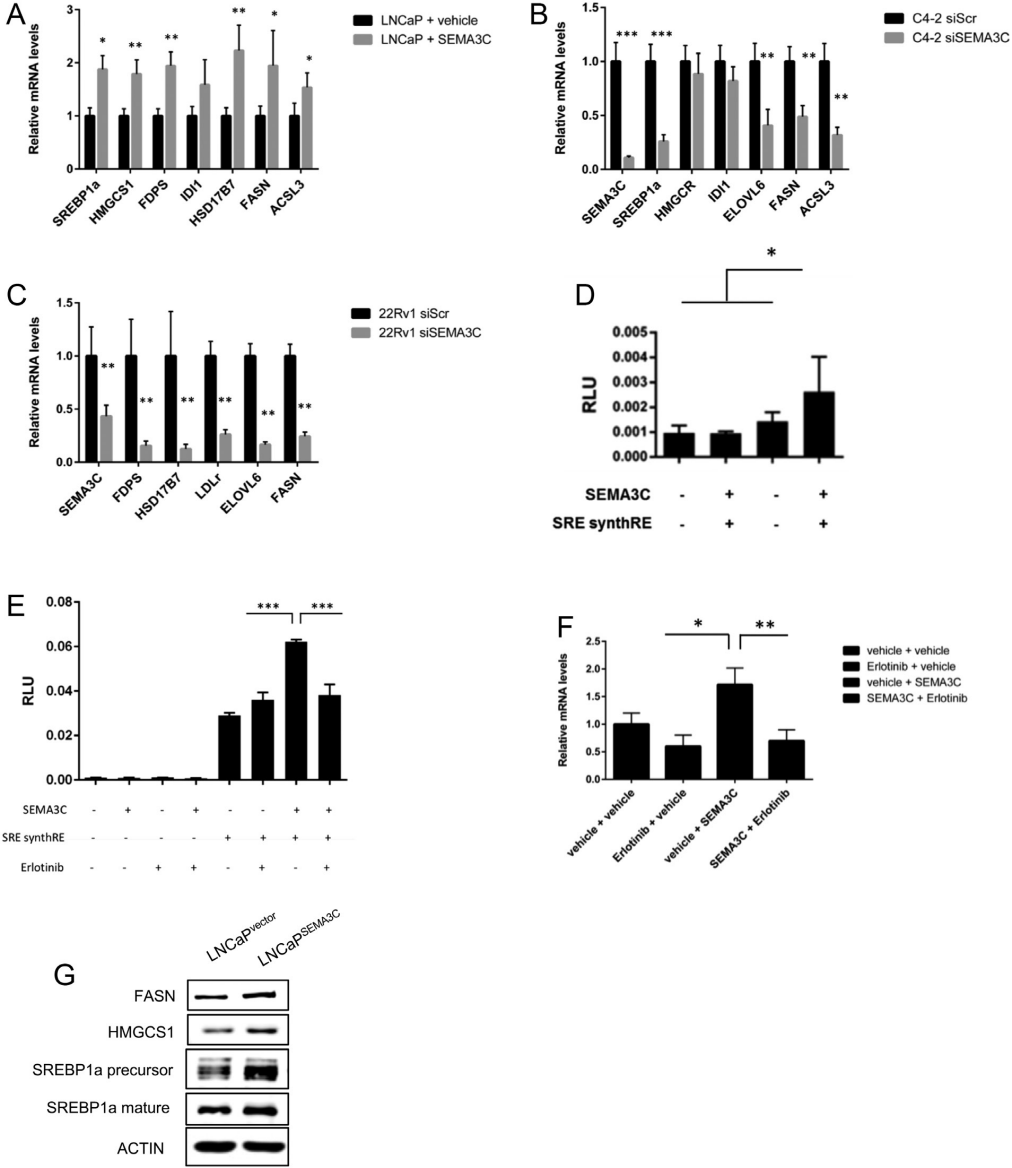
SEMA3C upregulates lipogenic and cholesterogenic enzymes. (A) LNCaP cells treated with recombinant SEMA3C 1 μM in 5% CSS-supplemented phenol red-free RPMI for 24 h. mRNA expression of genes involved in cholesterol pathway by RT-qPCR method. SEMA3C was silenced in (B) C4-2 cells and (C) 22Rv1 cells by siRNA and mRNA expression of cholesterogenic genes was quantified in siSEMA3C-treated cells compared to respective control cells. (D) Increased activity of SREBP-responsive element in transiently transfected luciferase reporter plasmid into LNCaP cells followed by recombinant SEMA3C 1 μM or vehicle treatment (*n* = 6). (E) LNCaP cells were treated with erlotinib 2 μM approximately 2 h prior to the treatment with recombinant SEMA3C 1 μM. After 24 h, SREBP-responsive promoter activity was assessed by luciferase assay. (F) qPCR was performed to quantify relative mRNA expression of a panel of SREBP targets in LNCaP cells in the presence of erlotinib 2 μM. Only HMGCS1 mRNA expression level was affected. (G) The whole protein of LNCaP^SEMA3C^ and LNCaP^vector^ was immunoblotted to examine the protein level of genes involved in the cholesterol pathway. (mean ± s.d.; *P < 0.05, **P < 0.01, ***P < 0.001).

Next, we tested if SEMA3C can regulate the expression of enzymes involved in the ^14^C-acetate metabolism. Treatment of LNCaP cells with recombinant SEMA3C (1 μM) induced mRNA expression of SREBP1a and its target genes (Horton *et al.* 2002) coding for multiple enzymes involved in the mevalonate pathway, 3-hydroxy-3-methylglutaryl-CoA synthase 1 (HMGCS1), farnesyl diphosphate synthase (FDPS), isopentenyl-diphosphate delta-isomerase (IDI1), HSD17B7, and also enzymes responsible for fatty acid metabolism, such as fatty acid synthase (FASN) and acyl-CoA synthetase long-chain family member 3 (ACSL3) (Fig. 5A). siRNA-mediated silencing of SEMA3C in C4-2 cells suppressed expression of SREBP1a, IDI1, FASN, and ACSL3 along with other targets 3-hydroxy-3-methylglutaryl-CoA reductase (HMGCR) and ELOVL fatty acid elongase 6 (ELOVL6) (Fig. 5B). Also, SEMA3C knockdown in 22Rv1 cells downregulated FDPS, HSD17B7, ELOVL6, FASN, and another SREBP target, LDLr, involved in cholesterol uptake (Fig. 5C). We confirmed the effect of SEMA3C on SREBP-mediated transcriptional activity by conducting a luciferase assay on LNCaP cells, which were transiently transfected with luciferase reporter plasmid harboring SREBP-responsive element. Recombinant SEMA3C treatment significantly increased luciferase activity compared to control cells (Fig. 5D). Erlotinib suppressed SEMA3C’s effect on SREBP transcriptional activity (Fig. 5E). Also, treating LNCaP cells with erlotinib reduced the induction of HMGCS1 by SEMA3C (Fig. 5F). These results suggest that SEMA3C may regulate the SREBP pathway by activating EGFR. Increased protein expression of SREBP1a and its targets, FASN and HMGCS1, in LNCaP^SEMA3C^ compared to control cells was confirmed by immunoblotting (Fig. 5G).

### SEMA3C altered expression of steroidogenic and cholesterogenic genes

**Figure 6:**
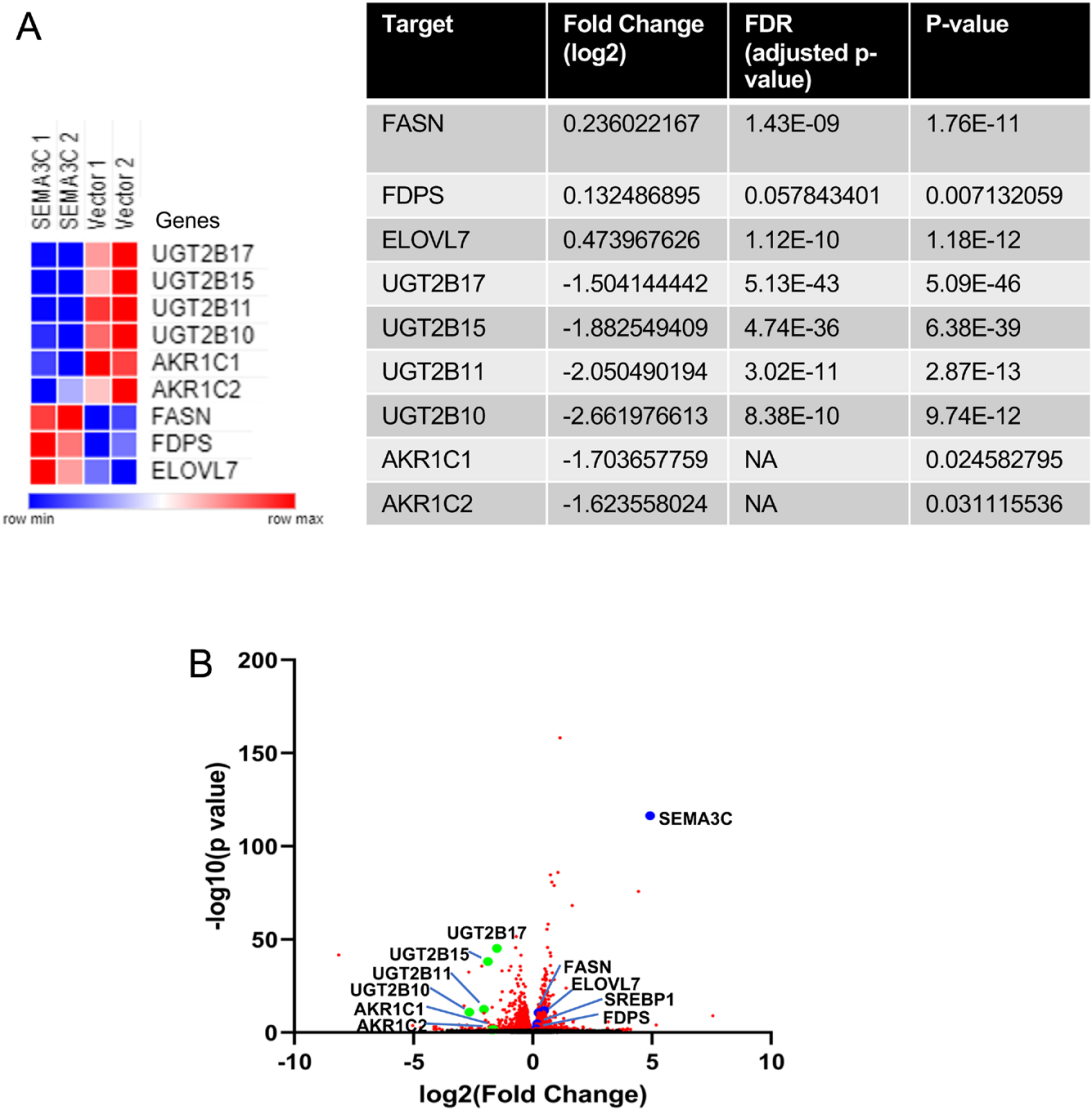
(A) Total RNA was isolated from LNCaP^SEMA3C^ and LNCaP^vector^ (*n* = 3 per group) to be analyzed by RNA-seq. Morpheus software (https://software.broadinstitute.org/morpheus/) was utilized to generate a heatmap for the visualization of differential gene expression. Mean values of respective gene expression from two biological replicates (SEMA3C 1, 2 and Vector 1, 2) were used to provide us with a heat map based on the matrix of values which is mapped to a matrix of colors. (B) A volcano plot was generated to show the statistical significance (*P*-value) vs the magnitude of change (fold change).

Effect of SEMA3C on the expression of enzymes involved in steroidogenesis pathway was confirmed by sequencing whole transcriptome of LNCaP^SEMA3C^ and LNCaP^vector^ cells. DEseq analysis showed that SEMA3C overexpression is correlated with the expression of multiple genes involved in steroidogenesis and cholesterol synthesis pathways. SEMA3C overexpression is associated with increased expression of SREBP targets, FASN, FDPS, and ELOVL7. However, there is a reverse correlation between expression of SEMA3C and androgen-catabolizing enzymes, AKR1C1, AKR1C2, UGT2B10, UGT2B11, UGT2B15, and UGT2B17 (Fig. 6A). Differential expression of these genes is displayed on a volcano plot (Fig. 6B).

### SEMA3C enhanced castration-resistant tumor growth through *de novo* steroidogenesis

**Figure 7:**
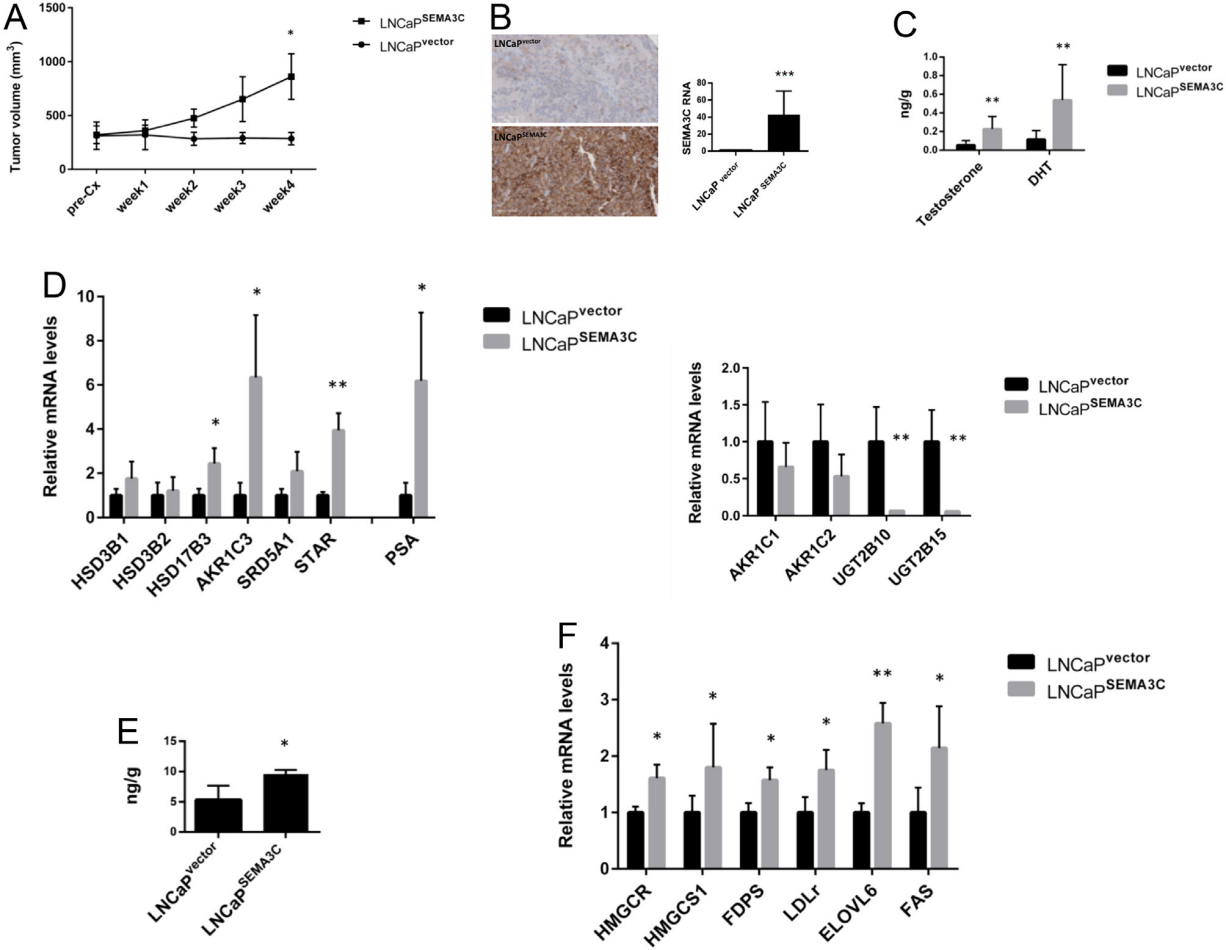
SEMA3C promotes intratumoral androgen synthesis. SEMA3C-overexpressing tumors were developed by inoculation of 1 × 10^6^ LNCaP^SEMA3C^ or LNCaP^vector^ in each of two injection sites of male nude mice (*n* = 6). (A) Tumor volumes (mm^3^) were measured weekly in both SEMA3C and control groups, before collection 4 weeks post castration (post-Cx). (B) SEMA3C overexpression in LNCaP^SEMA3C^ tumors was validated in RNA and protein levels by RT-qPCR and IHC methods. (C) LC-MS analysis of isolated steroids from both tumor types showed significant induction in testosterone and DHT production by LNCaP^SEMA3C^ tumor cells vs controls. (D) Whole RNA was isolated from tumor lysates, and mRNA expression of PSA and a panel of steroidogenic and androgen-catabolizing enzymes in LNCaP^SEMA3C^ tumors compared to LNCaP^vector^ tumors. (E) SEMA3C gene overexpression in LNCaP tumor cells increased intratumoral concentration of free cholesterol quantified by LC-MS technique (*n* = 6). (F) mRNA expression of genes coding for enzymes involved in cholesterol synthesis and uptake was quantified in LNCaP^SEMA3C^ compared to LNCaP^vector^ tumor cells (mean ± s.d.; *P < 0.05, **P < 0.01, ***P < 0.001).

SEMA3C overexpressing LNCaP xenograft tumors were developed by inoculating LNCaP^SEMA3C^ and LNCaP^vector^ cells in nude mice, as described in the Materials and methods section. LNCaP^SEMA3C^ xenografts had a higher tumor growth rate 4 weeks post castration than LNCaP^vector^ tumors (Fig. 7A). This result is consistent with Peacock *et al.* 2018 report, showing that SEMA3C overexpression in LNCaP xenograft tumor increases their growth rate post castration. We validated SEMA3C overexpression by IHC of the LNCaP^SEMA3C^ tumors harvested 4 weeks post castration (Fig. 7B). LC-MS analysis of steroids isolated from tumor homogenates showed that intratumoral concentration of testosterone and DHT significantly increased in LNCaP^SEMA3C^ tumors compared to LNCaP^vector^ tumors (Fig. 7C), consistent with the induction of key steroidogenic enzymes, STAR, HSD3B1, HSD3B2, AKR1C3, HSD17B3, and SRD5A1, along with the downregulation of androgen-inactivating enzymes AKR1C1, AKR1C2, UGT2B10, and UGT2B15 by SEMA3C. Increased PSA mRNA expression by SEMA3C suggests that SEMA3C overexpression induced PSA expression in the xenograft tumors (Fig. 7D). Also, LC-MS analysis of lipid extracts of tumor homogenates showed a statistically significant increase in free cholesterol level in LNCaP^SEMA3C^ tumor samples compared to control tumors (Fig. 7E). Also, SEMA3C overexpression affected intratumoral lipid concentration, whose increase did not reach statistical significance (data not shown). Consistent with *in vitro* results, SEMA3C induced mRNA expression of enzymes and proteins of the SREBP pathway in LNCaP xenograft tumor cells, HMGCR, HMGCS1, FDPS, LDLr, FASN, and ELOVL6 (Fig. 7F).

## Discussion

Intratumoral androgen synthesis is increasingly becoming recognized as a key mechanism underlying the resistance of prostate cancer cells to ADT (Stanbrough *et al.* 2006, Locke *et al.* 2009, Mostaghel *et al.* 2018). However, the cell-extrinsic factors that trigger the molecular mechanism(s) driving steroidogenesis in prostate cancer cells are poorly understood. Here, we demonstrate that SEMA3C, an autocrine prostate cancer growth factor, can induce the expression of key steroidogenic enzymes while simultaneously suppressing the expression of androgen-inactivating enzymes leading to increased synthesis of androgens such as testosterone and DHT from adrenal hormones such as DHEA or *de novo* from cholesterol. In addition, our findings suggest that SEMA3C mediates its effects on *de novo* steroidogenesis, in part, by activating the SREBP transcription factor. Our results suggest that SEMA3C may be acting through its ability to stimulate EGFR signaling since SEMA3C-induced steroidogenesis was suppressed by the presence of erlotinib.

RTK-activating growth factors are a class of cell-extrinsic factors that drive steroidogenesis. It has been demonstrated that IGF2 and insulin stimulate steroidogenesis in PCa cells (van der Veeken *et al.* 2009, Lubik *et al.* 2011, 2013) while EGF signaling has been shown to stimulate steroidogenesis in Leydig tumor cells (Cameron *et al.* 1996, Manna *et al.* 2002, Shiraishi & Ascoli 2006). Our finding that SEMA3C can stimulate steroidogenesis in PCa cells is consistent with these data, since we have previously shown that SEMA3C activates multiple RTKs, such as EGFR, HER2, and MET in a cognate-independent manner (Xu *et al.* 2017, Peacock *et al.* 2018) suggesting that SEMA3C may be stimulating steroidogenesis by activating RTKs. Notably, we showed that erlotinib (an inhibitor of EGFR and HER2) attenuates SEMA3C’s effects on the induction of cholesterogenic and steroidogenic enzymes in LNCaP cells suggesting that EGFR and HER2 may be the key RTKs mediating steroidogenesis in LNCaP cells. Since LNCaP cells are MET null, we cannot rule out a potential role for MET in the induction of steroidogenesis.

SEMA3C’s effects, which we observed, are comparable to the activation of *de novo* steroidogenesis by insulin (Lubik *et al.* 2011) and IGF2 (Lubik *et al.* 2013). Insulin and IGF2 induce the expression of key steroidogenic enzymes including StAR, CYP17A1, HSD3B2, HSD17B3, and SRD5A1, whose expression is also similarly regulated by SEMA3C. However, there are differences in the pattern of induction for steroidogenic enzymes by insulin, IGF, and SEMA3C. For instance, AKR1C3, whose expression level remains unchanged by insulin (Lubik *et al.* 2011), is induced by IGF2 (Lubik *et al.* 2013) and SEMA3C. Also, HSD3B1 and SRD5A2 are increased by SEMA3C but not affected by insulin (Lubik *et al.* 2011) and IGF2 (Lubik *et al.* 2013), whereas CYP11A1 is induced by insulin (Lubik *et al.* 2011) and IGF2 (Lubik *et al.* 2013) but not affected by SEMA3C. With respect to the production of testosterone, DHT, and androsterone, SEMA3C induced production of these androgens similar to that seen with insulin and IGF2. All these growth factors are capable of increasing the intracellular androgen concentrations that are sufficient to activate AR (Gregory *et al.* 2001, Titus *et al.* 2005, Locke *et al.* 2008).

SEMA3C is a growth factor that can activate a number of RTKs including EGFR, HER2, and MET in a cognate ligand-independent manner. EGF (Stocco *et al.* 2005) similar to other growth factors such as insulin and IGF2 is able to stimulate steroidogenesis.Lubik *et al.* (2011) found that insulin treatment of prostate cancer cells upregulates steroidogenic enzymes. Also, insulin increases the synthesis of androgens, comparable to the levels reported in PCa patients. Furthermore,Lubik *et al.* (2013) reported that IGF2 affects the steroidogenesis pathway in a similar fashion (Lubik *et al.* 2013).

RTK signaling may induce steroidogenesis through various mechanisms. One potential mechanism by which RTK signaling can induce steroidogenesis is through the activation of SREBP by MAPK, a downstream kinase activated by RTK signaling. MAPK has been shown to stimulate SREBP activity (Kotzka *et al.* 2000, Roth *et al.* 2000, Kotzka *et al.* 2004) and, in turn, SREBP activity has been shown to regulate steroidogenesis (Shimizu-Albergine *et al.* 2016). Consistent with this hypothesis, we have found that SEMA3C increases SREBP mRNA expression and transcriptional activity, and this increased SREBP1 activity was associated with increased MAPK phosphorylation. Similarly, Lubik *et al.* also found that SREBP mRNA expression was also induced by insulin (Lubik *et al.* 2011). These data suggest that SEMA3C may stimulate steroidogenesis in part through the activation of SREBP.

One of the SREBP targets affected by SEMA3C, ACSL3, has comparable effects on steroidogenesis as SEMA3C. Migita *et al*. (2017) showed that ACSL3 overexpression in LNCaP cells significantly increased the expression of steroidogenic genes, AKR1C3 and HSD3B1. In parallel, ACSL3 overexpression significantly suppressed the expression of UGT2B15 and UGT2B17 involved in the DHT inactivation process. Also, ACSL3 overexpression in LNCaP cells is associated with the synthesis of testosterone and DHT in the presence of DHEAS (Migita *et al.* 2017). Intriguingly, SEMA3C upregulates ACSL3 in LNCaP cells, and SEMA3C siRNA knockdown in C4-2 cells significantly suppressed ACSL3 expression. Hence, SEMA3C-regulated ACSL3 may contribute to SEMA3C’s effects on the expression of steroidogenic enzymes. Further studies need to be conducted to reveal the molecular details of this association.

Here, we suggest that activation of RTK signaling pathways by SEMA3C may support PCa progression by promoting cell survival and growth and by stimulating intratumoral steroidogenesis. Herein, we identify SEMA3C as a potential therapeutic target in PCa. Blocking SEMA3C in combination with steroidogenesis inhibitors, such as abiraterone (Attard *et al.* 2008, 2011), may be a therapeutic opportunity to suppress intratumoral androgen biosynthesis during ADT to improve CRPC patient outcomes.

## Supplementary Materials

Supplementary Materials

Supplementary Figures

## Declaration of interest

The authors declare that there is no conflict of interest that could be perceived as prejudicing the impartiality of the research reported.

## Funding

This work was funded by grants from grants from Terry Fox Research Institute http://dx.doi.org/10.13039/501100004376 (TFRI #1109), US Department of Defense (DoD #W81XWH-21-1-0300), Prostate Cancer Canada http://dx.doi.org/10.13039/501100000109 (Grants #TAG2014-06 and GS2015-06), Michael Smith Foundation for Health Research http://dx.doi.org/10.13039/100005622 (Grant # 17318), Terry Fox Foundation http://dx.doi.org/10.13039/501100002655 (TFRI #1062), Cancer Research Society http://dx.doi.org/10.13039/100009326 (Grant #F09-60564), the NIH Pacific Northwest Prostate SPORE (NCI P50 CA097186), Prostate Cancer Foundation http://dx.doi.org/10.13039/100000892 British Columbia (Grant #F13-0016), and National Centres of Excellence of Canada (CECR PC-TRIADD).
