# Supplementary material for: Semaphorin 3C promotes de novo steroidogenesis in prostate cancer cells
Source: Endocr Relat Cancer. 2023 Nov 6;30(12):e230010. doi: 10.1530/ERC-23-0010 (PMC10692650; doi:10.1530/ERC-23-0010)
Supplement: Supplementary Materials [file supplementary_materials.pdf]

Table of Contents

Table of Contents **1**

Appendix Materials and Methods **3**

Analysis of intracellular and secreted steroids **3**

Radiometric analysis of steroidogenesis **3**

Lipid extraction from tumor tissue **4**

Appendix Table S1 **5**

Appendix Figure S1 **7**

Appendix Figure S2 **11**

Appendix Figure S3 **14**

Appendix Materials and Methods

Analysis of intracellular and secreted steroids

Steroids were extracted from cell pellets and tissue homogenates with hexane/ethyl acetate (Hex/EtOAc, 60/40, v/v). Steroids in media were isolated with solid phase extraction (SPE) columns (Waters C18 SepPak 50mg) conditioned with 1ml acetonitrile, equilibrated with 1ml water prior to sample loading, followed by a 1ml water wash before eluting with 1ml acetone. Internal standard (d3T and d3DHT) was added to each sample prior to extraction. Extracts were dried down using a CentriVap centrifugal evaporation system (35ºC) and residues redissolved in 50mM hydroxylamine in 50% MeOH by vortexing and sonication, and the resulting solutions heated for 1h in a 65ºC water bath. The extracted/derivatized samples were analysed using a Waters Acquity Liquid Chromatography system and Waters Quattro Premier XEtandem mass spectrometer with MassLynx™ 4.1 (Waters) for instrument control. Separations were carried out with a 2.1x100mm BEH 1.7µM C18 column, mobile phase water (A) and 0.1% formic acid in acetonitrile (B) (gradient: 0.2 min, 25% B; 8min, 70% B; 9 min, 100% B; 12 min 100% B; 12.2 min, 20% B; 14 min run length). Column temperature was 40ºC and injection volumes were 15 µl. The MS was set slightly below unit resolution for enhanced sensitivity, capillary was 3.0 kV, source and desolvation temperatures were 120°C and 350°C respectively, desolvation and cone gas flows were 1000 L/hr and 50 L/hr, collision cell pressure was held at 6.4e-3 mbar. All data was collected in ES+ mode using multiple reaction monitoring (MRM) with cone voltage and collision energies optimized for each analyte from scan and fragment scan analysis of derivatized standards. Area under curve (AUC) of analyte versus internal standard was used for quantitation. Matrix free calibration standards from 0.01 up to 50 ng/ml were used as no suitable blank matrix was possible for homogenates and little to no matrix effect observed with media. Deuterated internal standards d3T and d3DHT were used against all steroids with final concentrations of 0.4 and 1.8ng/ml respectively. QuanLynx Software (Waters) was used to integrate chromatographic traces and date exported to Excel for further normalizing as needed.

Radiometric analysis of steroidogenesis

$LNCaP_{\mathbf{SEMA3C/vector}}$ cells were cultured in six-well plates in full growing media as previously stated. To begin the experiment, cells were dosed with 6 µCi/ml ^{14}C-acetate (PerkinElmer catalog number NEC553050UC) in phenol red-free RPMI with 5% (v/v) CSS for 72 h; subsequently, conditioned media was examined for presence of ^{14}C-labaled analytes. Secreted steroids were isolated with SPE columns as previously described. Dried down samples were resuspended in 75 µl 50% methanol, and analyzed on a Waters 2695 Separations Module connected to a Radiomatic 150TR Flow Scintillation Analyzer equipped with a 0.5mL flow cell. LC flow rate was 0.3mL/min, column temperature was 30ºC and Radiomatic™ scintillation fluid (Ultima Flo M, Perkin Elmer) flow rate was 1mL/min. The ^{14}C-labaled steroids were separated on a Waters 2.1X150mm 5µm, XTerra MX C18 column equilibrated with 10:90 acetonitrile (ACN):H_2O, followed by a gradient to 25:75 ACN:H_2O from 0.75-1.5 min, further to 35:65 ACN:H_2O from

1.5-20 min, and to 45:55 ACN:H_2O from 25-30 min. Isopropanol (IPA) was introduced at this time to a final 45:0:55 ACN:IPA:H_2O from 30-50 min, retained at 45:55:0 until 55 min and back to starting conditions at 57 min. for re-equilibration for final run length 70 min..^{3}H-DHT, ^{3}H-progesterone and ^{14}C-cholesterol were applied as radiometric retention time (RT) standards. Additional nonlabelled (cold) standards were used to determine the delay of radiometric RTs compared to MS RT by ~1 min and this normalization factor was useful to correlate the ^{14}C-analytes peaks with nonlabeled steroid standards.

Lipid extraction from tumor tissue

Tumor tissue homogenates, 10ul each sample, were mixed and vortexed with 200ul methanol (MeOH) spiked with 20ul of 50µg/ml internal standard d6-6α-hydroxycholestanol (d6C). After adding 900ul Methyl tert-butyl ether (MTBE), the mixtures were vortexed in room temperature for 30min. Next, ddH_2O 500ul was added and samples were centrifuged at room temperature for 10 min to layer separation. The organic layer was collected and dried using Centrivap at 40^{oc} for 20min. To derivatize cholesterol, dried extracts were first dissolved and vortexed in 400 ul Acetyl Cl/CHCl3 v/v (1:5) at room temperature for 1 hour, and then dried by Centrivap in 40^{oc} for ~15min, and re-dissolved in 300 ul MeOH/CHCl3 v/v 70:30. CHCl3 was diluted with MeOH to v/v 10% and final concentration of d6C was 1µg/ml. Cholesterol concentration measured by LCMS was normalized to weight of the 10ul tumor homogenate was used for lipid extraction. Cholesteryl ester mobile phase consisted of acetonitrile/0.1M ammonium acetate 9/1 (A) and isopropanol (B) with the following gradient: 0.2min, 25%B; 5-8min, 70%B, 8.1min, 25% B with a 10min run length. Instrument parameters were optimized for the m/z's of ammonium adducts of acetate derivatized cholesterol and cholesterol oleate and their common m/z369 fragment was used for MRM quantitation. A linear calibration curve from 0.2-10µg/ml, R^2>0.99, was generated by derivatizing cholesterol/cholesterol oleate with d6C as IS, encompassing all data points. Recoveries and conversions to derivatized species were greater than 80% for steroids, 90% for cholesterol and 75% for cholesterol oleate and levels of steroids cholesteryl esters are normalized to initial tumor weight.

Gene	qPCR primers forward and reverse 5' to 3' sequences
GAPDH	CACCAGGGCTGCTTTTAACTC<br>GACAAGCTTCCCGTTCTCAG
RPL32	CCCCTTGTGAAGCCCAAGA<br>GACTGGTGCCGGATGAACTT
SEMA3C	GACAATTTGCGTGTTGGTTG<br>CGGTCCTGATCTTCATCCA
STAR	CAGTGGGTGCCTTCCAGAAATA<br>TGACTGGTGCCTATGAAAGCAA
CYP11A1	CTGCATCTTCAGTCGTCTGTCC<br>GGTGACCACTGAGAACCCATTC
CYP17A1	TCCCCAAGGTGGTCTTTCTGAT<br>GTGGACAGGGGCTGTGAGTTAC
HSD3B1	CCATGTGGTTTGCTGTTACCAA<br>TCAAAACGACCCTCAAGTTAAAAGA
HSD3B2	CTGCTGCCTCTCTTTCACACAA<br>AGAAAGTTCTGGTTGGGCCAGT
AKR1C1	CCTAAAAGTAAAGCTTTAGAGGCCACC<br>GAAAATGAATAAGGTAGAGGTCAACATAAT
AKR1C2	CCTAAAAGTAAAGCTCTAGAGGCCGT<br>GAAAATGAATAAGATAGAGGTCAACATAG
AKR1C3	GAGAAGTAAAGCTTTGGAGGTCACA<br>CAACCTGCTCCTCATTATTGTATAAATGA
HSD17B2	CCAGGGAAAGGCGCTTACTTGT<br>GGGCTTGTCTTGGCCAAAATGT
HSD17B3	CTGAAGCTCAACACCAAGGTCA<br>CTGCTCCTCTGGTCCTCTTCAG
HSD17B4	GACTACGCCAAGCTCTGAAGG<br>AAGCAATTTCCCCTGCATCTT
SRD5A1	CCTGTTGAATGCTTCATGACTTG<br>TAAGGCAAAGCAATGCCAGATG
SRD5A2	CTCTCTAAGGAAGGGGCCGAAC<br>GACAATGCATTCCGCAAACATA
UGT2B10	TGACATCGTTTTTGCAGATGCTTA<br>CATTGTCTCAAATAATGTAGTG

UGT2B15	TGGGAATATTATGACTACAG<br>AGGGGTTTGGCTGGTTTAC
FKBP5	TCCCTCGAATGCAACTCTCT<br>GCCACATCTCTGCAGTCAAA
PSA	ACCAGAGGAGTTCTTGACCCCAAA<br>CCCCAGAATCACCCGAGCAG
TMPRSS2	CCATTTGCAGGATCCGTCTG<br>GGATGTGTCTTGGGGAGCAA
ELOVL6	CAAAGCACCCGAACTAGGAG<br>TGGTGATACCAGTGCAGGAA
FAS	CTTCCGAGATTCCATCCTACGC<br>TGGCAGTCAGGCTCACAAACG
FDPS	AAAGCAGGATTTCGTTCAGCA<br>GGAATGCTACTACCACCGTCAA
HMGCR	GGATGACTCGTGGCCCAGT<br>TCGAGCCAGGCTTTCACTTC
HMGCS1	TTCGTGGCTCACTCCCTTT<br>CTGTCACTGTTTCCTCCTTCG
HSD17B7	AACAGGAACTTCAACCAGCAG<br>GGCATCAACAGCGTCCATA
IDI1	GCTAGGAATTCCCTTGGAAGA<br>GTTCACCCCAGATACCATCAG
LDLr	CTACAAGTGGGTCTGCGATG<br>TTTGCAGGTGACAGACAAGC
SREBP1	CGCTCCTCCATCAATGACAA<br>TCGAGAAAGCGAATGTAGTCGAT
SREBP2	TGAAGCTGGCCAATCAGAAAA<br>AGTCTGGTGGACAGTGATGTGG

Appendix Table S1. Sequences of primers to conduct qPCR for specific targets.

a

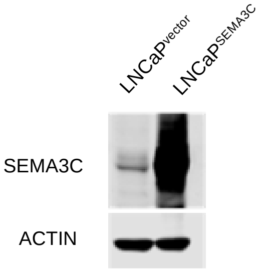

LNCaPvector
LNCaPSEMA3C
SEMA3C
ACTIN

b

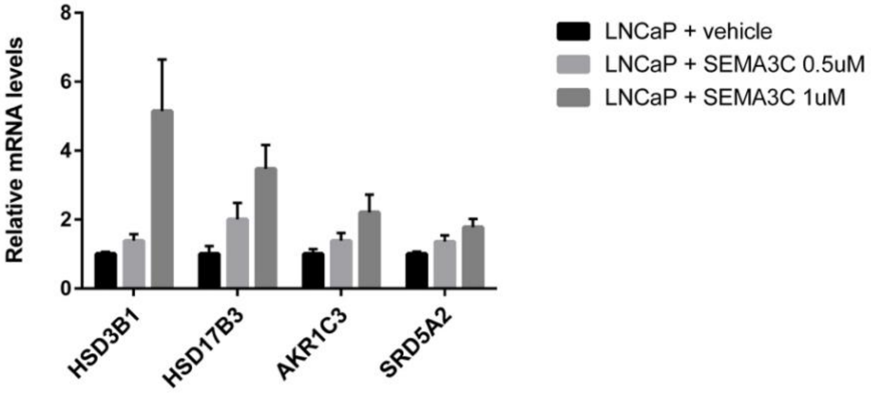

Relative mRNA levels
8
6
4
2
0
HSD3B1
HSD17B3
AKR1C3
SRD5A2
LNCaP + vehicle
LNCaP + SEMA3C 0.5uM
LNCaP + SEMA3C 1uM

c

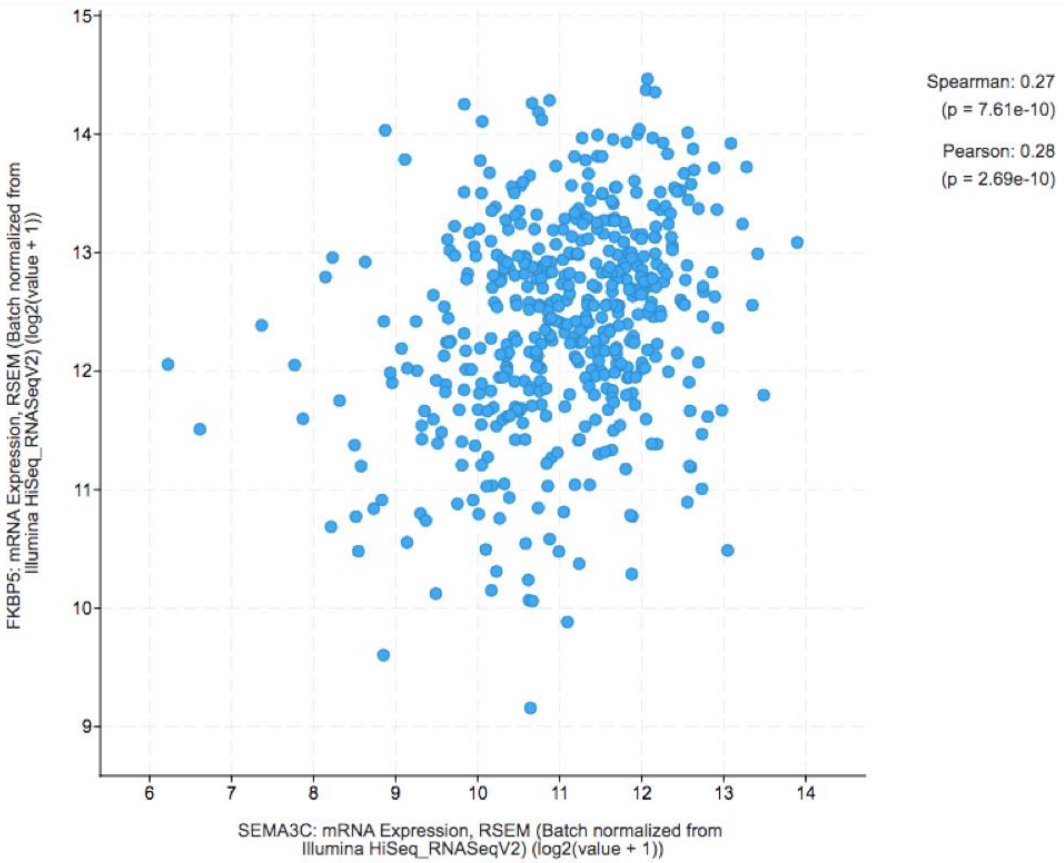

FKBP5: mRNA Expression, RSEM (Batch normalized from Illumina HiSeq_RNASeqV2) (log2(value + 1))
SEMA3C: mRNA Expression, RSEM (Batch normalized from Illumina HiSeq_RNASeqV2) (log2(value + 1))
Spearman: 0.27
(p = 7.61e-10)
Pearson: 0.28
(p = 2.69e-10)

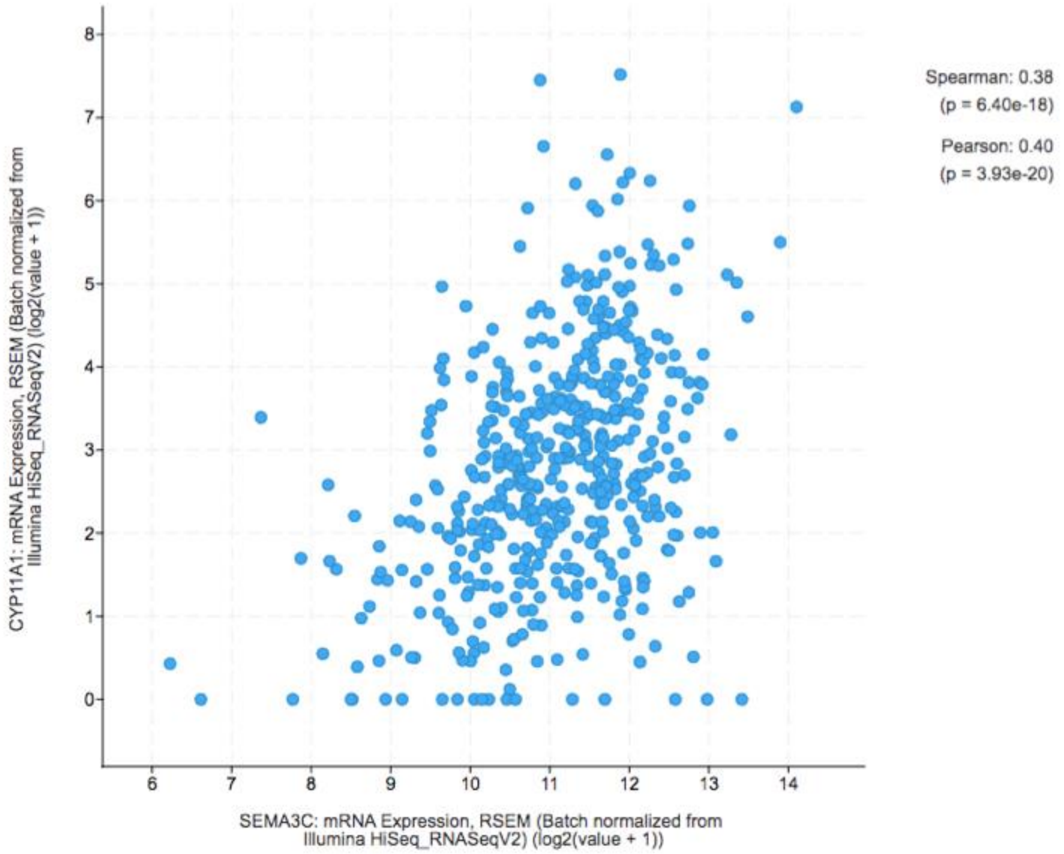
Spearman: 0.38
(p = 6.40e-18)
Pearson: 0.40
(p = 3.93e-20)
CYP11A1: mRNA Expression, RSEM (Batch normalized from Illumina HiSeq_RNASeqV2) (log2(value + 1))
SEMA3C: mRNA Expression, RSEM (Batch normalized from Illumina HiSeq_RNASeqV2) (log2(value + 1))

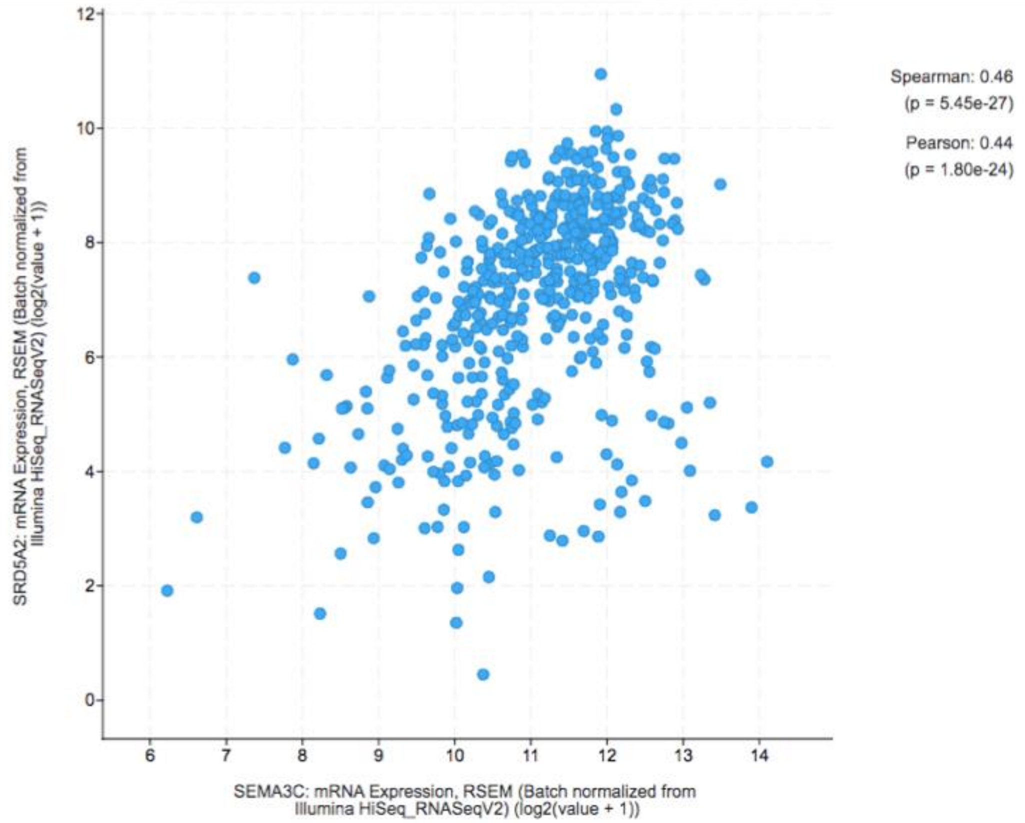
Spearman: 0.46
(p = 5.45e-27)
Pearson: 0.44
(p = 1.80e-24)
SRD5A2: mRNA Expression, RSEM (Batch normalized from Illumina HiSeq_RNASeqV2) (log2(value + 1))
SEMA3C: mRNA Expression, RSEM (Batch normalized from Illumina HiSeq_RNASeqV2) (log2(value + 1))

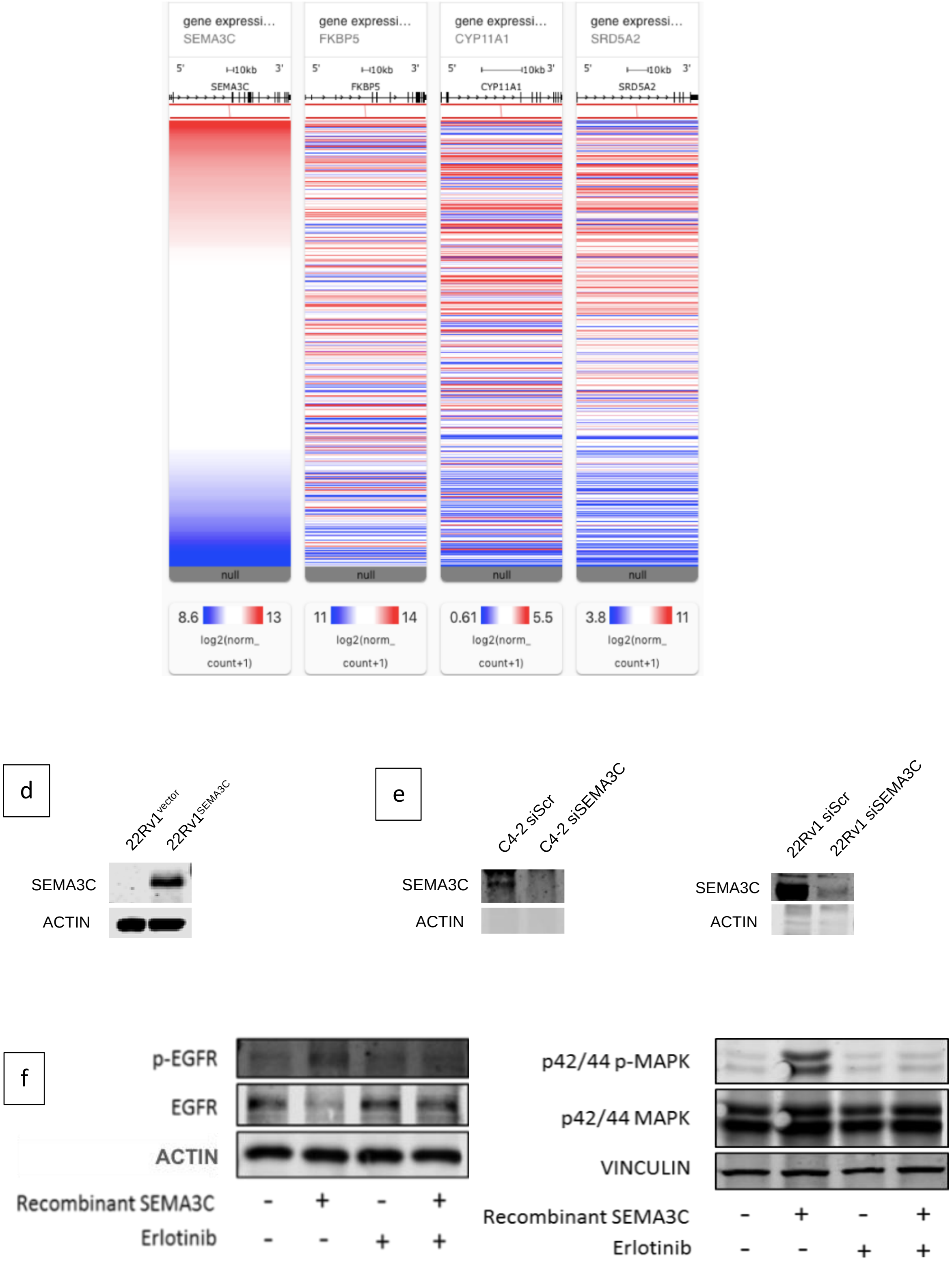

gene expressi...
SEMA3C
gene expressi...
FKBP5
gene expressi...
CYP11A1
gene expressi...
SRD5A2
5'
3'
10kb
null
8.6
13
11
14
0.61
5.5
3.8
11
log2(norm_
count+1)
d
22Rv1 vector
22Rv1 SEMA3C
SEMA3C
ACTIN
e
C4-2 siScr
C4-2 siSEMA3C
22Rv1 siScr
22Rv1 siSEMA3C
SEMA3C
ACTIN
f
p-EGFR
EGFR
ACTIN
Recombinant SEMA3C
Erlotinib
p42/44 p-MAPK
p42/44 MAPK
VINCULIN
Recombinant SEMA3C
Erlotinib

Appendix Figure S1. **(a)** SEMA3C overexpression in LNCaP cells was validated by immunoblotting of 50 ug protein lysate of SEMA3C-overexpressing LNCaP cells for SEMA3C. SEMA3C protein level in LNCaP^{SEMA3C} was compared to that in empty LNCaP cells. **(b)** LNCaP cells were treated with vehicle or recombinant SEMA3C 0.5 and 1 uM for 24 hours. mRNA expression of steroidogenic enzymes was quantified by qPCR. (Mean ± SD; *P<0.05) **(c)** cBioPortal plots of the TCGA provisional dataset (https://www.cbioportal.org/) shows the degree of correlation in expression of SEMA3C and each of enzymes CYP11A1 and SRD5A2. UCSC Xena visualization software (https://xena.ucsc.edu/) was used to examine co-expression of SEMA3C and steroidogenic enzymes, CYP11A1 and SRD5A2, in the TCGA PRAD prostate cancer dataset. Fold-changes are shown in colour, log2 (normalized_count+1) mean is subtracted per column across 550 samples. **(d)** SEMA3C overexpression in 22Rv1 cells was validated by immunoblotting of 50 ug protein lysate of SEMA3C-overexpressing 22Rv1 cells for SEMA3C, comparing the SEMA3C protein level between 22Rv1^{SEMA3C} and control 22Rv1 cells. **(e)** Reduced protein level of SEMA3C in C4-2 and 22Rv1 cells treated with siSEMA3C was compared to SEMA3C protein expression in respective control cells. **(f)** The effect of erlotinib on EGFR signaling was confirmed with immunoblotting for pEGFR, EGFR, p42/44 p-MAPK, and p42/44 MAPK.

a

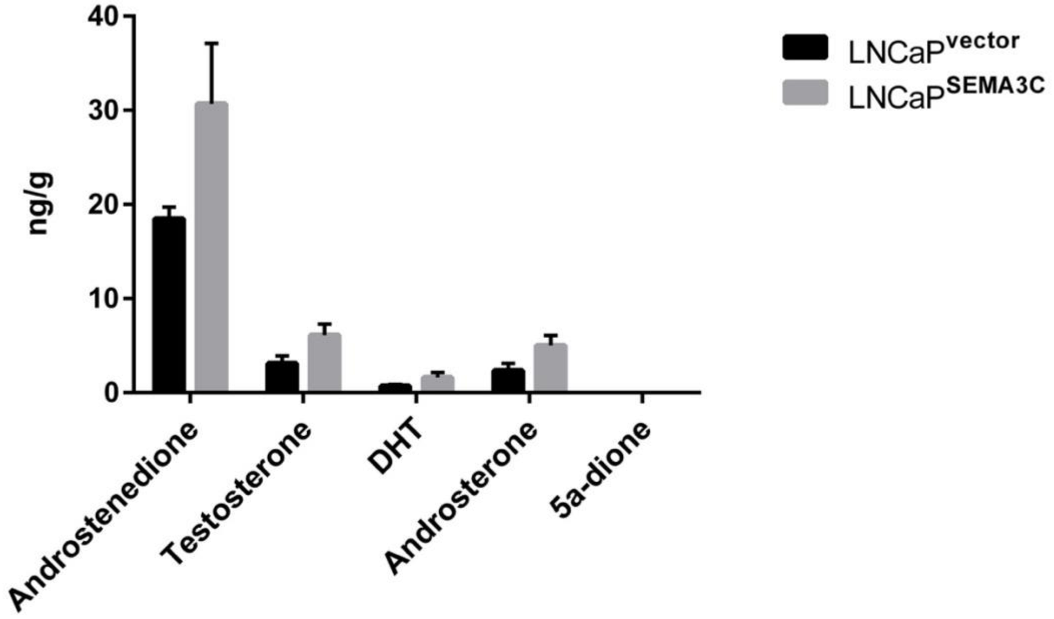

40
30
20
10
0
ng/g
Androstenedione
Testosterone
DHT
Androsterone
5a-dione
LNCaP vector
LNCaP SEMA3C

b

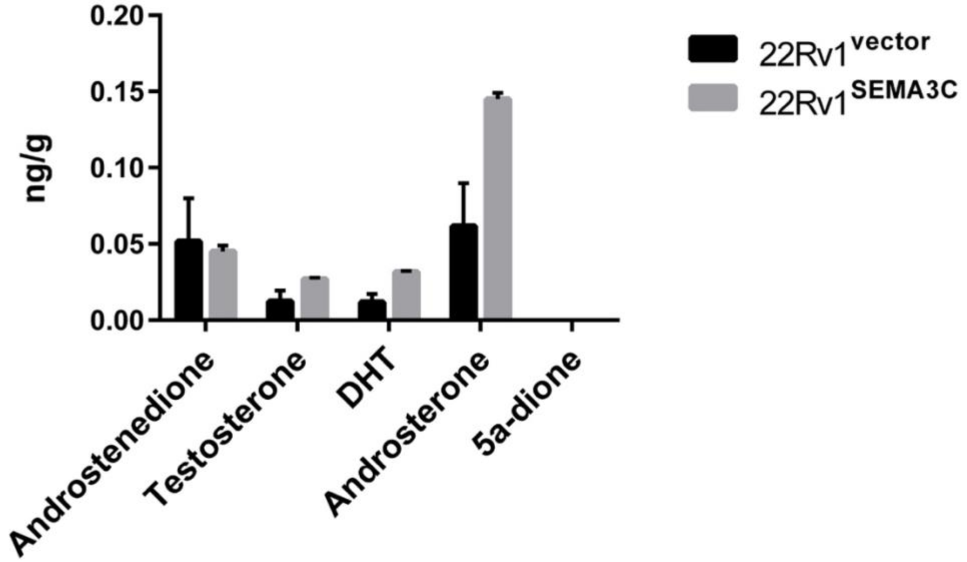

0.20
0.15
0.10
0.05
0.00
ng/g
Androstenedione
Testosterone
DHT
Androsterone
5a-dione
22Rv1 vector
22Rv1 SEMA3C

c

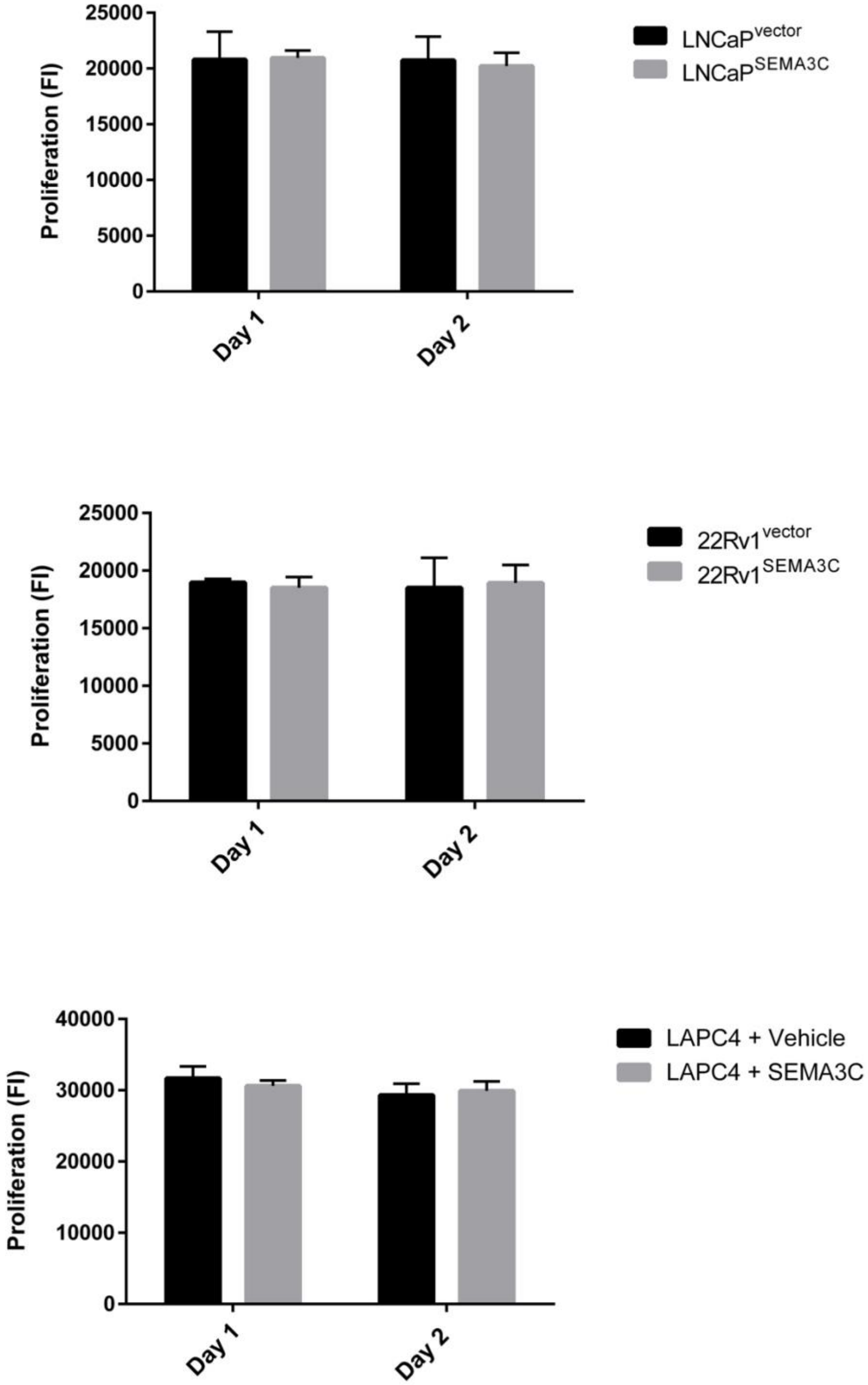

Proliferation (FI)
25000
20000
15000
10000
5000
0
Day 1
Day 2
LNCaP^{vector}
LNCaP^{SEMA3C}
Proliferation (FI)
25000
20000
15000
10000
5000
0
Day 1
Day 2
22Rv1^{vector}
22Rv1^{SEMA3C}
Proliferation (FI)
40000
30000
20000
10000
0
Day 1
Day 2
LAPC4 + Vehicle
LAPC4 + SEMA3C

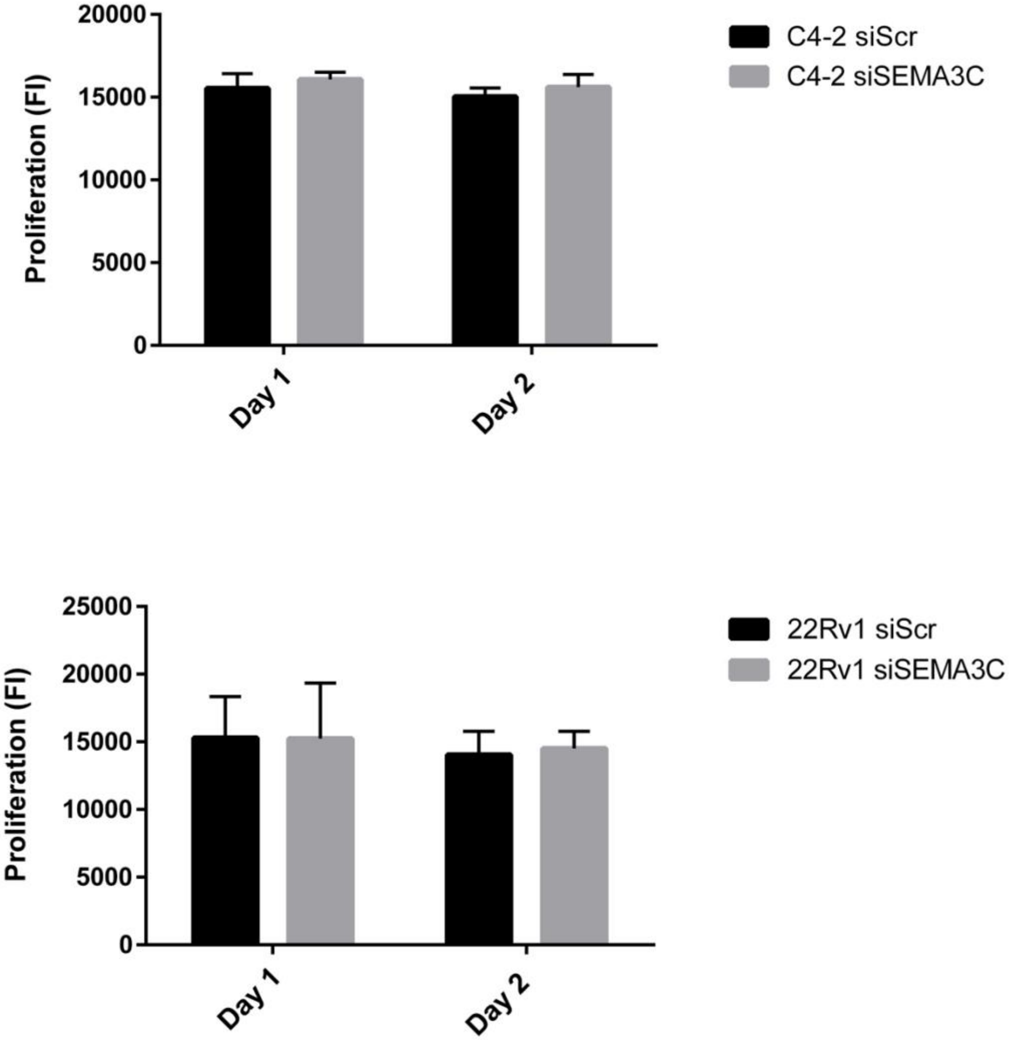

Appendix Figure S2. Intracellular steroids isolated from **(a)** SEMA3C-overexpressing LNCaP and **(b)** SEMA3C-overexpressing 22Rv1 were compared to respective control cells. **(c)** Proliferation of $\text{LNCaP}^{\text{SEMA3C}}$ *vs.* $\text{LNCaP}^{\text{vector}}$ cells, $\text{22Rv1}^{\text{SEMA3C}}$ *vs.* $\text{22Rv1}^{\text{vector}}$ cells, recombinant SEMA3C-treated *vs.* vehicle-treated LAPC4 cells, 22Rv1 transfected with siSEMA3C compared to siScr control cells, and C4-2 transfected with siSEMA3C compared to siScr control cells was assessed with PrestoBlue assay. Following incubation, cells were stained with PrestoBlue reagent %10 (v/v) in 24 and 48-hour time points. Data represent the mean ± STDEV of four replicates.

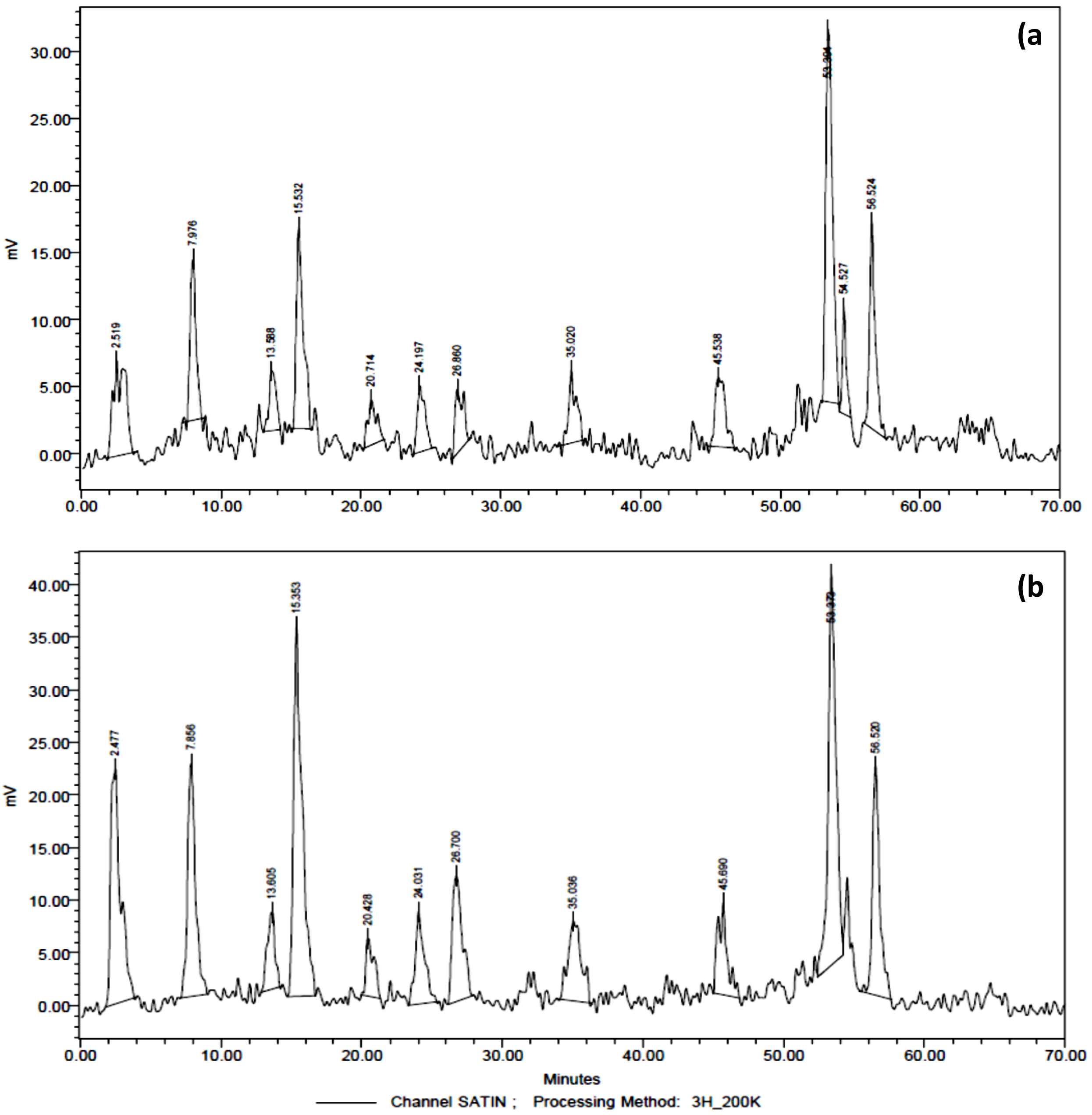

Appendix Figure S3. SEMA3C gene overexpression increases de novo steroidogenic capacity of LNCaP cells. **(a)** LNCaP$_{\mathbf{vector}}$ and **(b)** LNCaP$_{\mathbf{SEMA3C}}$ were cultured in 10% FBS RPMI media, and next day changed into 5% CSS red phenol free RPMI media supplemented with 6uCi/ml $^{\mathbf{C14}}$acetate for 72 hours. Secreted steroids in cell media were isolated with SPE columns and radiolabeled steroids were identified and quantified by HPLC coupled with radiometric Scintillation Analyzer. Radioisotope-labeled controls including cholesterol, pregnenolone, DHT, and also nonlabelled (cold) standards were used to identify the labeled steroids synthesized by LNCaP cells based on the MS retention time agreements with controls: $^{\mathbf{C14}}$pregnenolone (~26.7 minutes), $^{\mathbf{C14}}$testosterone (~15.3 minutes), and $^{\mathbf{C14}}$DHT (~20.5 minutes).
