# Supplementary material for: Semaphorin 3C promotes de novo steroidogenesis in prostate cancer cells
Source: Endocr Relat Cancer. 2023 Nov 6;30(12):e230010. doi: 10.1530/ERC-23-0010 (PMC10692650; doi:10.1530/ERC-23-0010)
Supplement: Supplementary Figures [file supplementary_figures.pdf]

Full images of immunoblots

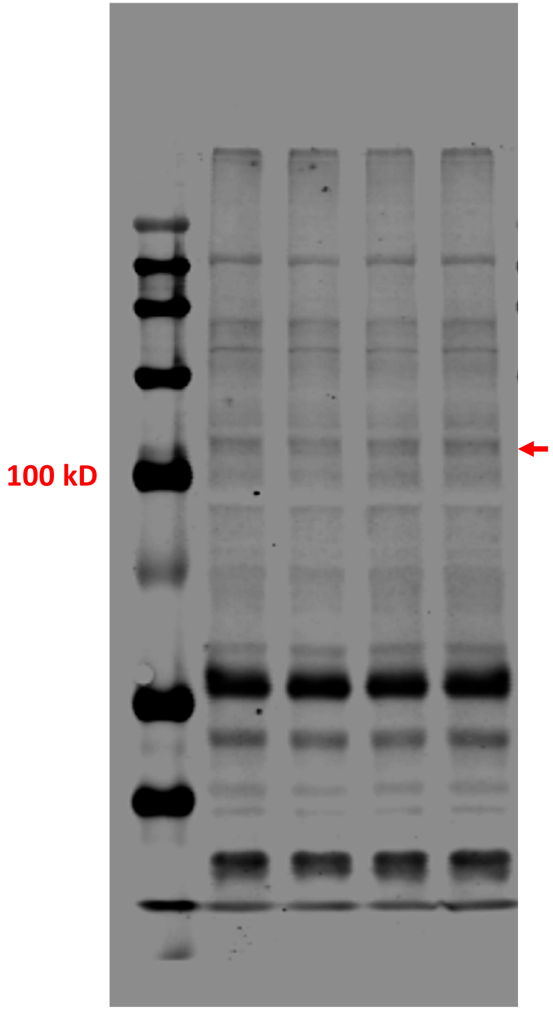

AR

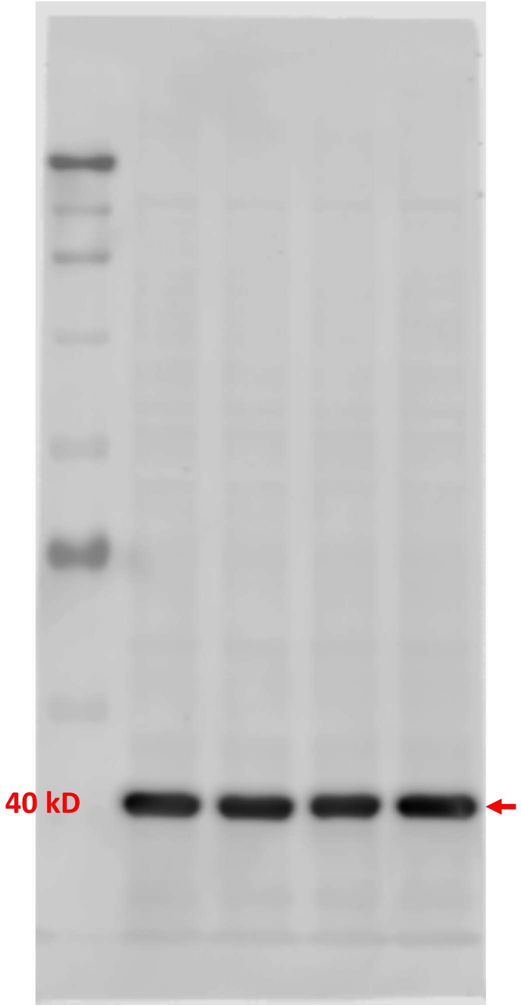

ACTIN

Immunoblotting for AR (110 kD) and ACTIN (40 kD). These images were cropped to be presented in **Figure 3C**.

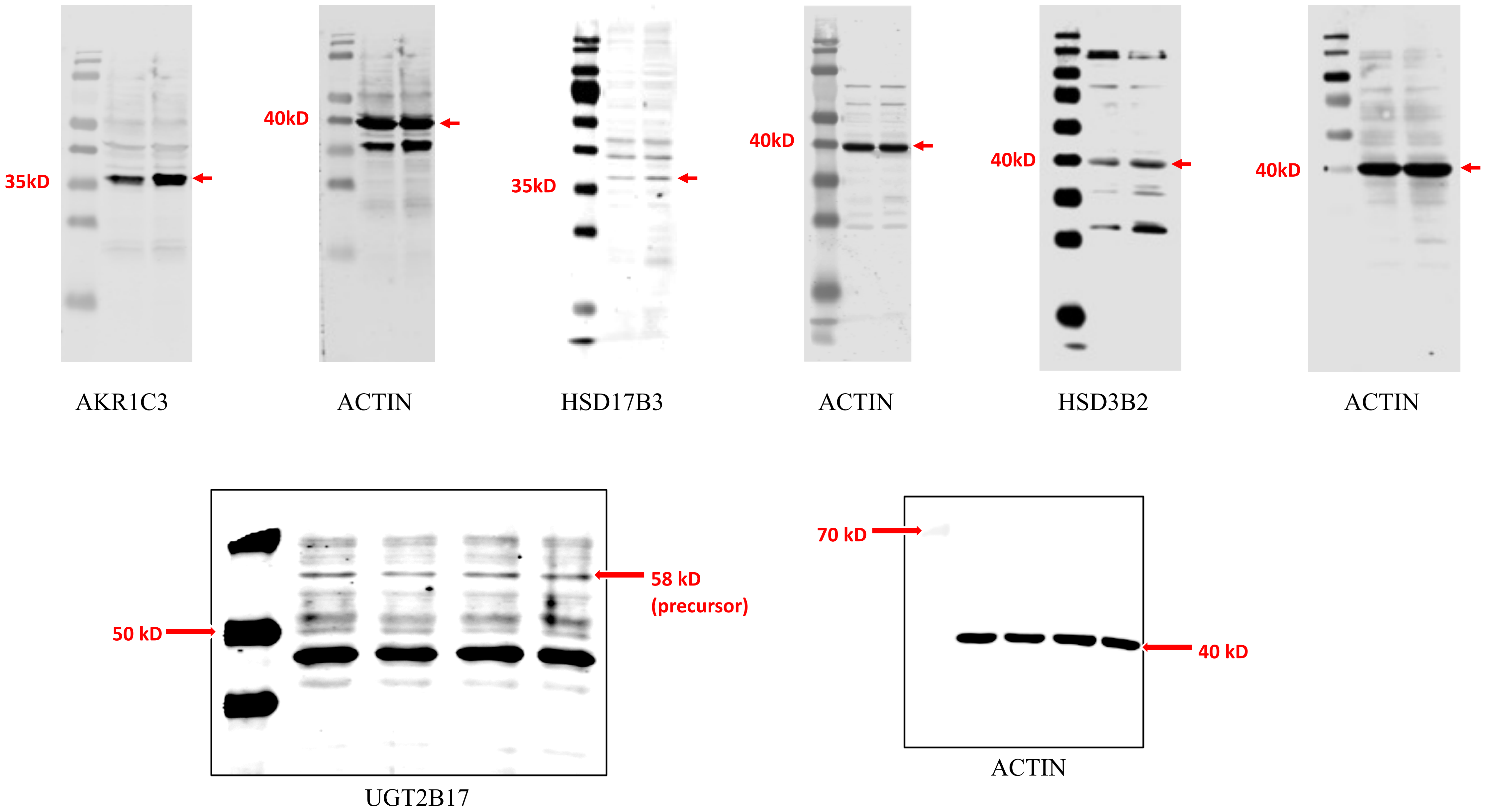

Immunoblotting for steroidogenic enzymes AKR1C3 (36 kD). HSD17B3 (35 kD), HSD3B2 (37 kD), UGT2B17 (58 kD) and ACTIN (40 KD). These images were cropped to be presented in **Figure 4B**.

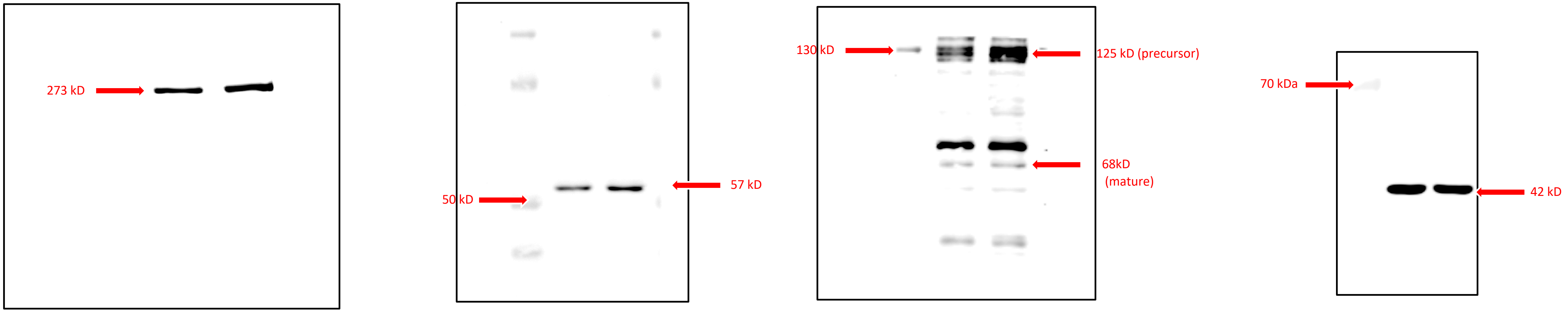

FASN HMGCS1 SREBP1a ACTIN

Immunoblotting for cholesterogenic enzymes FASN, HMGCS1, SREBP1a and ACTIN. These images were cropped to be presented in **Figure 5G**.

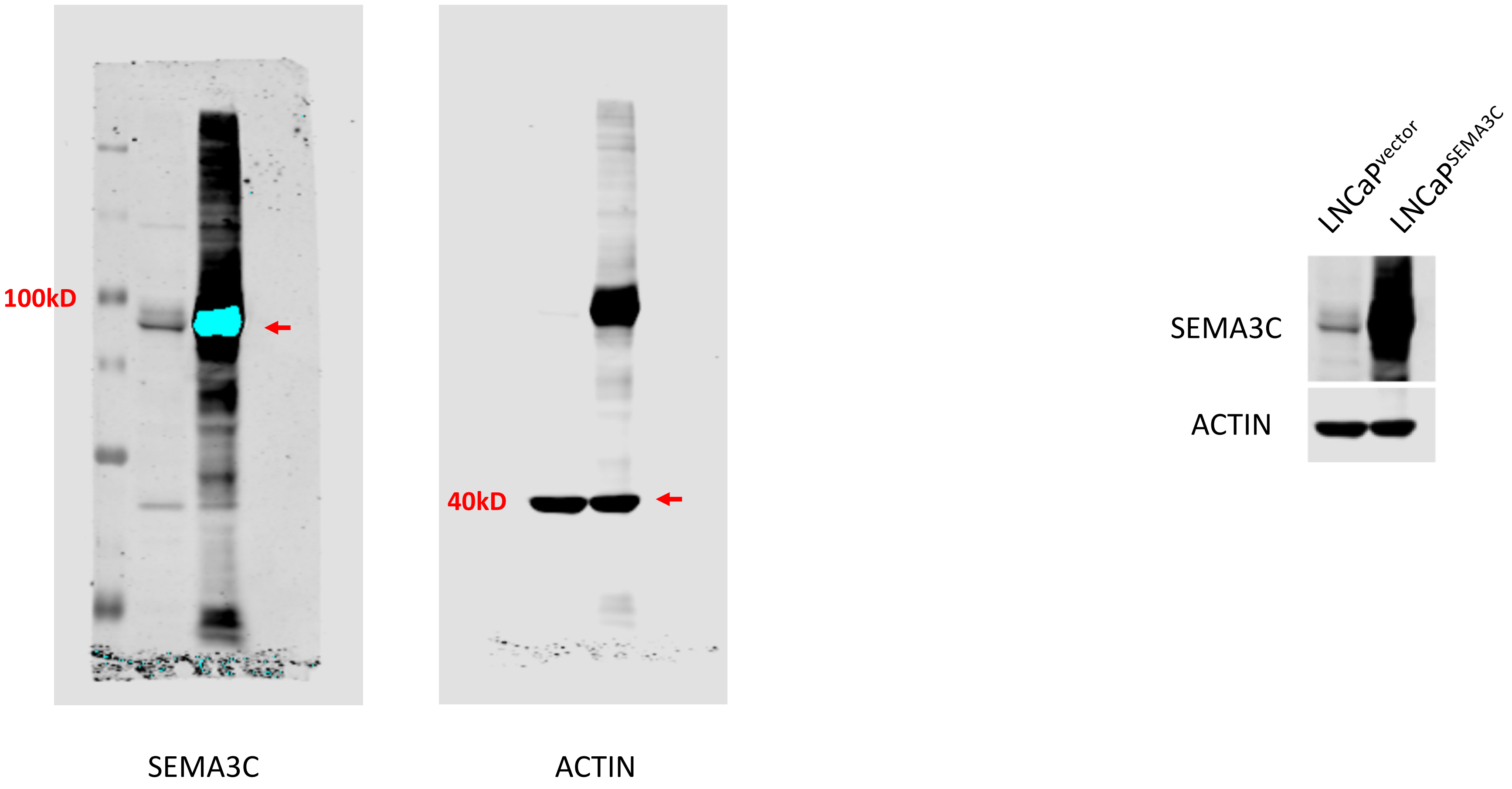

Immunoblotting for SEMA3C (96KD) and ACTIN (40KD). These images were cropped to be presented in **Appendix Figure S1a**.

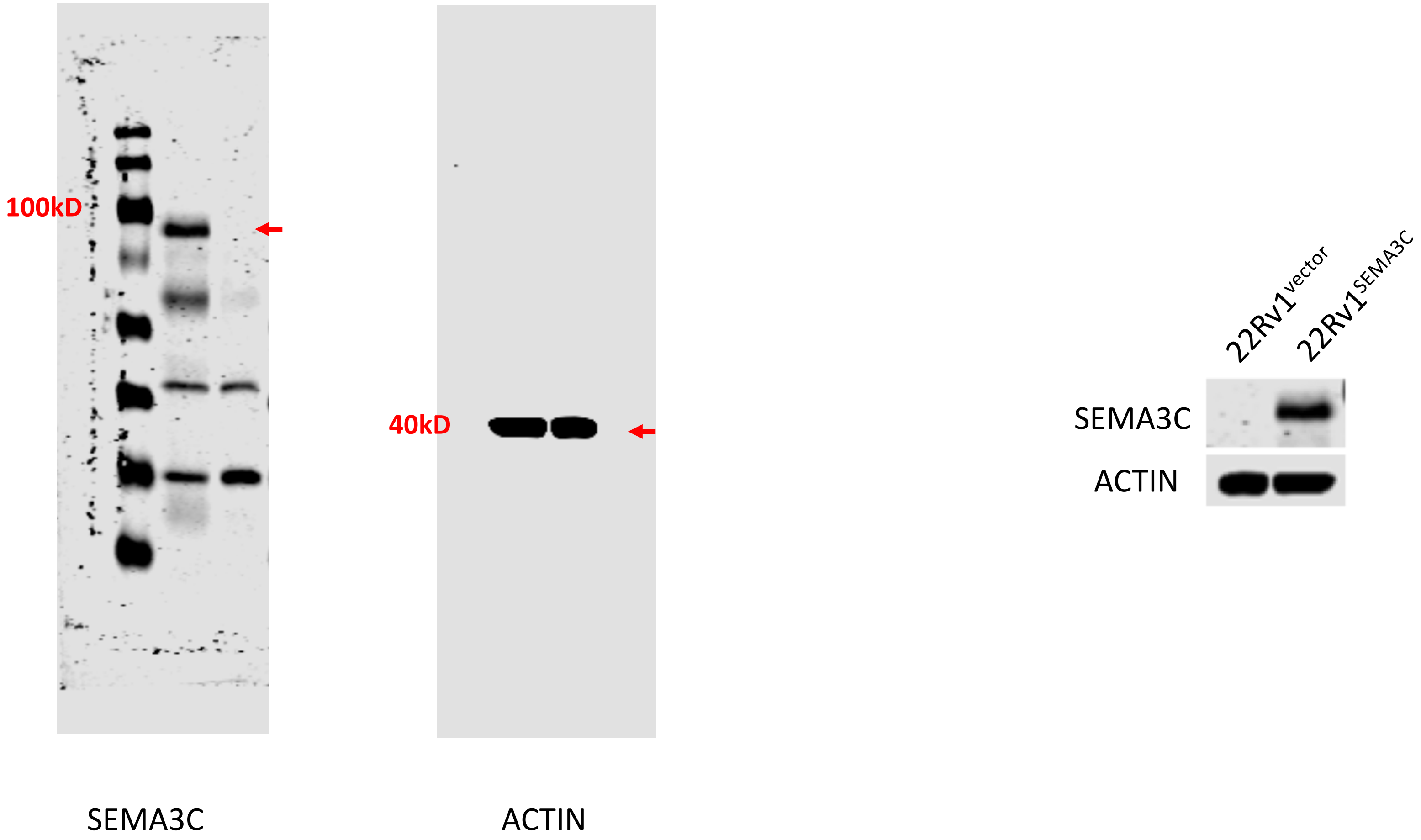

Immunoblotting for SEMA3C (96kD) and ACTIN (40kD). These images were cropped to be presented in **Appendix Figure S1d**.

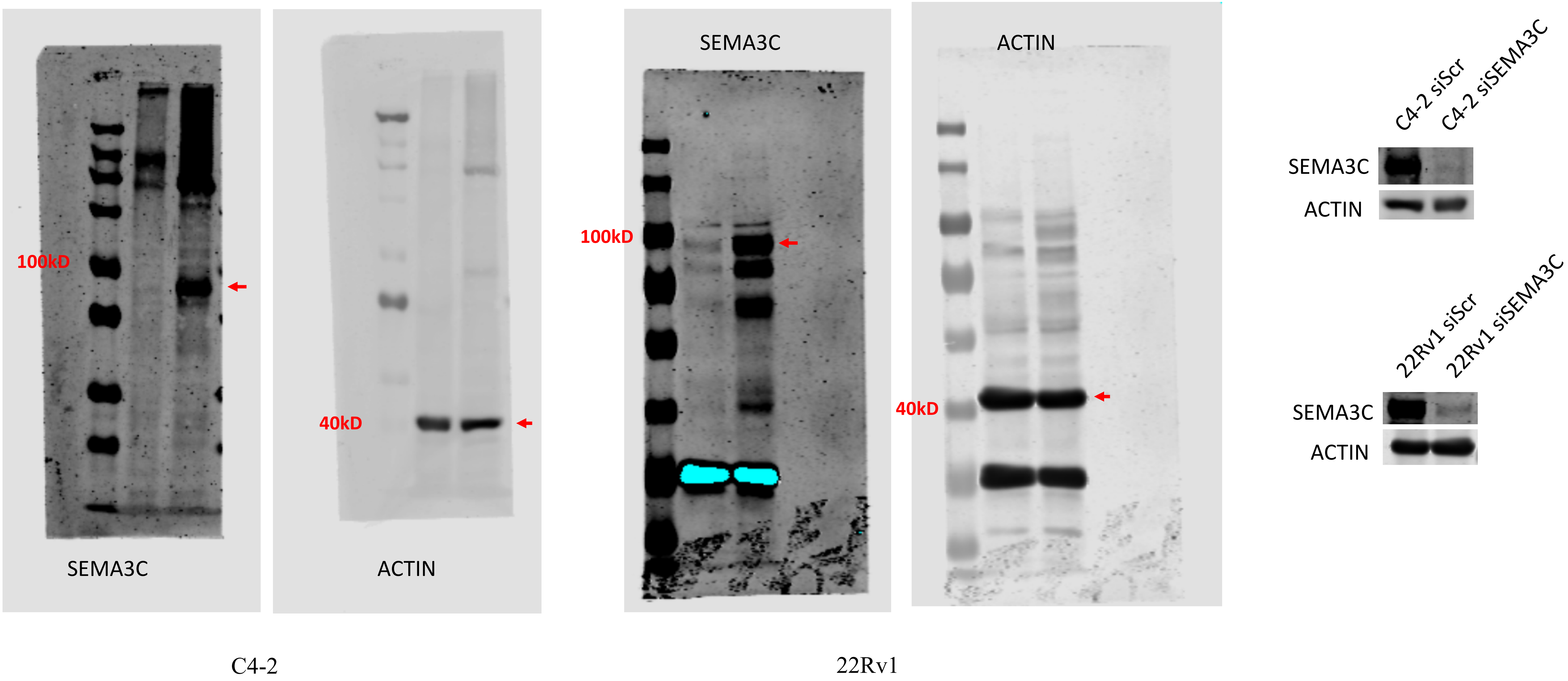

Immunoblotting for SEMA3C (96kD) and ACTIN (40kD). These images were cropped to be presented in **Appendix Figure S1e**.

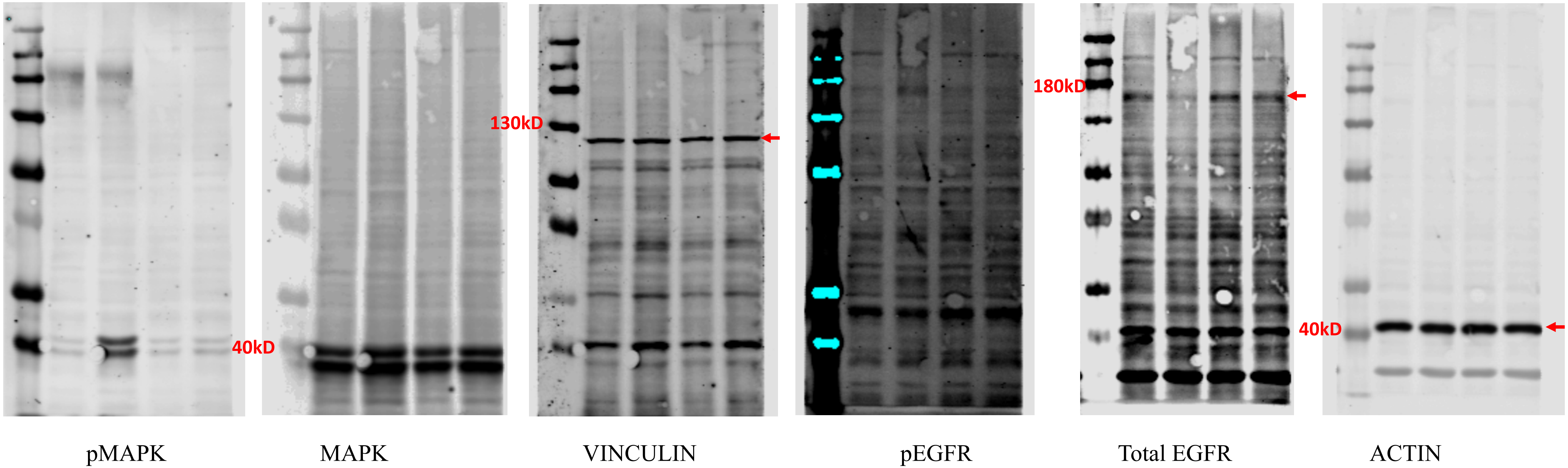

Immunoblotting for MAPK (40kD), EGFR (175kD), VINCULIN (124kD) and ACTIN (40kD). These images were cropped to be presented in **Appendix Figure S1f**.
